# Polysaccharide Nanocrystals-Based Chiral Nematic Structures: From Self-Assembly Mechanisms, Regulation, to Applications

**DOI:** 10.1021/acsnano.4c03130

**Published:** 2024-08-13

**Authors:** Huan Liu, Zhihao Wang, Haowei Xin, Jun Liu, Qianqian Wang, Bo Pang, Kai Zhang

**Affiliations:** † Biofuels Institute, School of the Environment and Safety Engineering, School of Emergency Management, Jiangsu University, Zhenjiang 212013, China; ‡ National Forestry and Grassland Administration Key Laboratory of Plant Fiber Functional Materials, Fuzhou 350108, China; § Department of Food Science and Technology, National University of Singapore, 2 Science Drive 2, Singapore, 117542, Singapore; ∥ Department of Materials and Environmental Chemistry, Stockholm University, Stockholm 10691, Sweden; ⊥ Sustainable Materials and Chemistry, Department of Wood Technology and Wood-Based Composites, University of Göttingen, Göttingen 37077, Germany

**Keywords:** polysaccharide nanocrystals, cellulose nanocrystals, chitin nanocrystals, self-assembly, chiral nematic structure, mechanism, regulation, applications

## Abstract

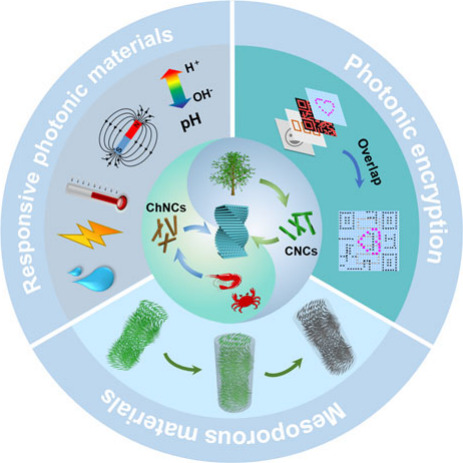

Chiral architectures, one of the key structural features of natural systems ranging from the nanoscale to macroscale, are an infinite source of inspiration for functional materials. Researchers have been, and still are, strongly pursuing the goal of constructing such structures with renewable and sustainable building blocks via simple and efficient strategies. With the merits of high sustainability, renewability, and the ability to self-assemble into chiral nematic structures in aqueous suspensions that can be preserved in the solid state, polysaccharide nanocrystals (PNs) including cellulose nanocrystals (CNCs) and chitin nanocrystals (ChNCs) offer opportunities to reach the target. We herein provide a comprehensive review that focuses on the development of CNCs and ChNCs for the use in advanced functional materials. First, the introduction of CNCs and ChNCs, and cellulose- and chitin-formed chiral nematic organizations in the natural world, are given. Then, the self-assembly process of such PNs and the factors influencing this process are comprehensively discussed. After that, we showcased the emerging applications of the self-assembled chiral nematic structures of CNCs and ChNCs. Finally, this review concludes with perspectives on the challenges and opportunities in this field.

## 1 Introduction

Nature, as the ever-greatest magician, has created numerous materials with intricate structures in elegant ways. Chiral structure is one of the most fascinating architectures, well-developed in various natural systems spanning from subnanoscale to galactic scale, such as angstrom-sized neutrino and amino acids, nanosized proteins, DNA, and polysaccharides, micrometer-scale bacteria, macroscopic living systems (e.g., seashell, plants, and horns), and even our light-year scale galaxy.^1−3^ Notably, such a structure is closely related to the physical, chemical, and biological properties of its chiral counterparts, especially those at the molecular and supramolecular levels. For instance, isomers of chiral small molecule drugs generally exhibit different biological activities and pharmacological functions, although they share the same chemical formula.^4^ The chirality of the sugars found in a hairpin-structured RNA minihelix (l-ribose or d-ribose) is the key factor that determines whether the amino acids loaded onto the RNA are left- or right-handed.^5^ Learning nature’s design principles involved in these fascinating structures offers a possibility for constructing functional materials.

**Figure 1:**
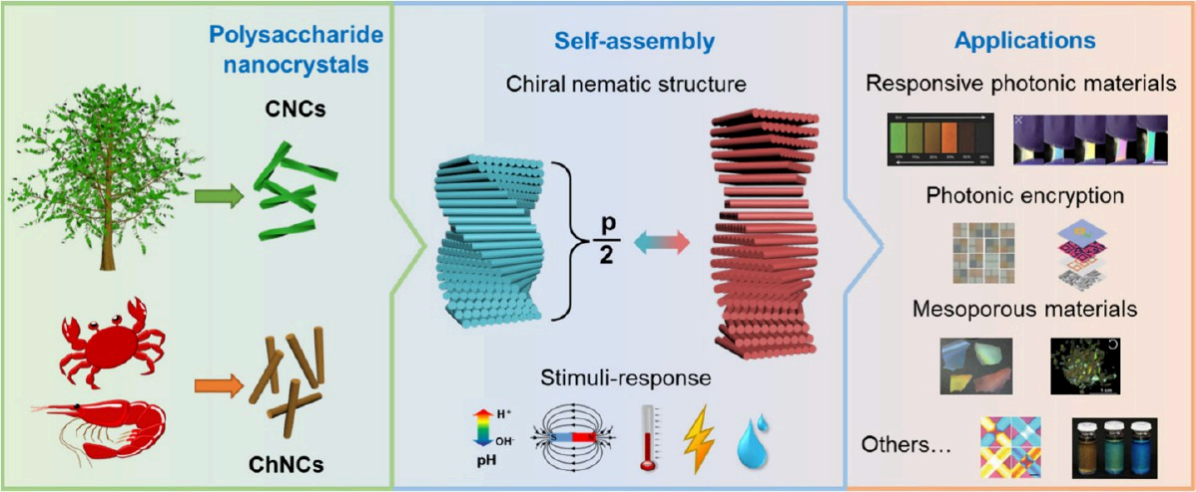
Schematic diagram showing the main contents of this review from the introduction and self-assembly of PNs to the potential applications of the self-assembled materials. Responsive photonic materials. Reprinted with permission from ref (6). Copyright 2017 Wiley-VCH. Reprinted with permission under a Creative Commons Attribution (CC BY) License from ref (7). Copyright 2019 Springer Nature. Photonic encryption. Reprinted with permission from refs (8) and (9). Copyright 2020 Wiley-VCH. Copyright 2023 Wiley-VCH. Mesoporous materials. Reprinted with permission from refs (10) and (11). Copyright 2010 Springer Nature. Copyright 2012 Wiley-VCH. Others. Reprinted with permission from ref (12). Copyright 2020 Wiley-VCH. Reprinted with permission under a Creative Commons Attribution (CC BY) License from ref (13). Copyright 2022 Springer Nature.

Among these chiral systems, chiral architectures composed of various polysaccharides, in particular cellulose and chitin, provide excellent paradigms for disclosing their formation mechanism and developing chiral artificial materials considering their inherent merits, such as easy availability, low cost, and high abundance.^14,15^ Currently, various top-down, bottom-up self-assemblies, and combinations of both approaches have been explored for the manufacture of polysaccharide-based chiral structures. Compared to top-down fabrication approaches, the bottom-up self-assembly strategy using polysaccharides nanocrystals (PNs), in particular, cellulose nanocrystals (CNCs) and chitin nanocrystals (ChNCs) as nanobuilding blocks, has established itself as the leading approach for fabricating chiral materials because of its nanoscale precision, and high time- and cost-efficiency.^16^ Rapid development has been witnessed in the realm of self-assembly of PNs regarding the elucidation of their self-assembly mechanism and the exploration of their applications over the past two decades (Figure 1).^17−29^ These studies greatly promote the development of chiral materials, including but certainly not limited to those derived from polysaccharides, thus requiring comprehensive analysis to identify future challenges and opportunities.

Several recent articles have reviewed the self-assembly of CNCs, with most of them focusing on their optical applications.^18,30−32^ However, to the best of our knowledge, very few articles have summarized and systematically discussed the self-assembly of polysaccharide nanocrystals containing CNCs and ChNCs in a comprehensive scope. Thus, in this review, we began with the introduction of CNCs and ChNCs followed by the chiral nematic structures in nature. We then comprehensively discussed the mechanism of the self-assembly of CNCs and ChNCs, and the regulation methods. Next, applications of the self-assembled materials were introduced. Finally, we present our viewpoints on some challenges that need to be further addressed in the area of PNs self-assembly. The purpose of this review is to offer perspectives on the development of PNs-based self-assembled functional materials with diverse possibilities in chiral photonic crystals, sensors, and other highly adaptable multifunctional systems.

### 1.1 Introduction to Cellulose Nanocrystals (CNCs)

**Figure 2:**
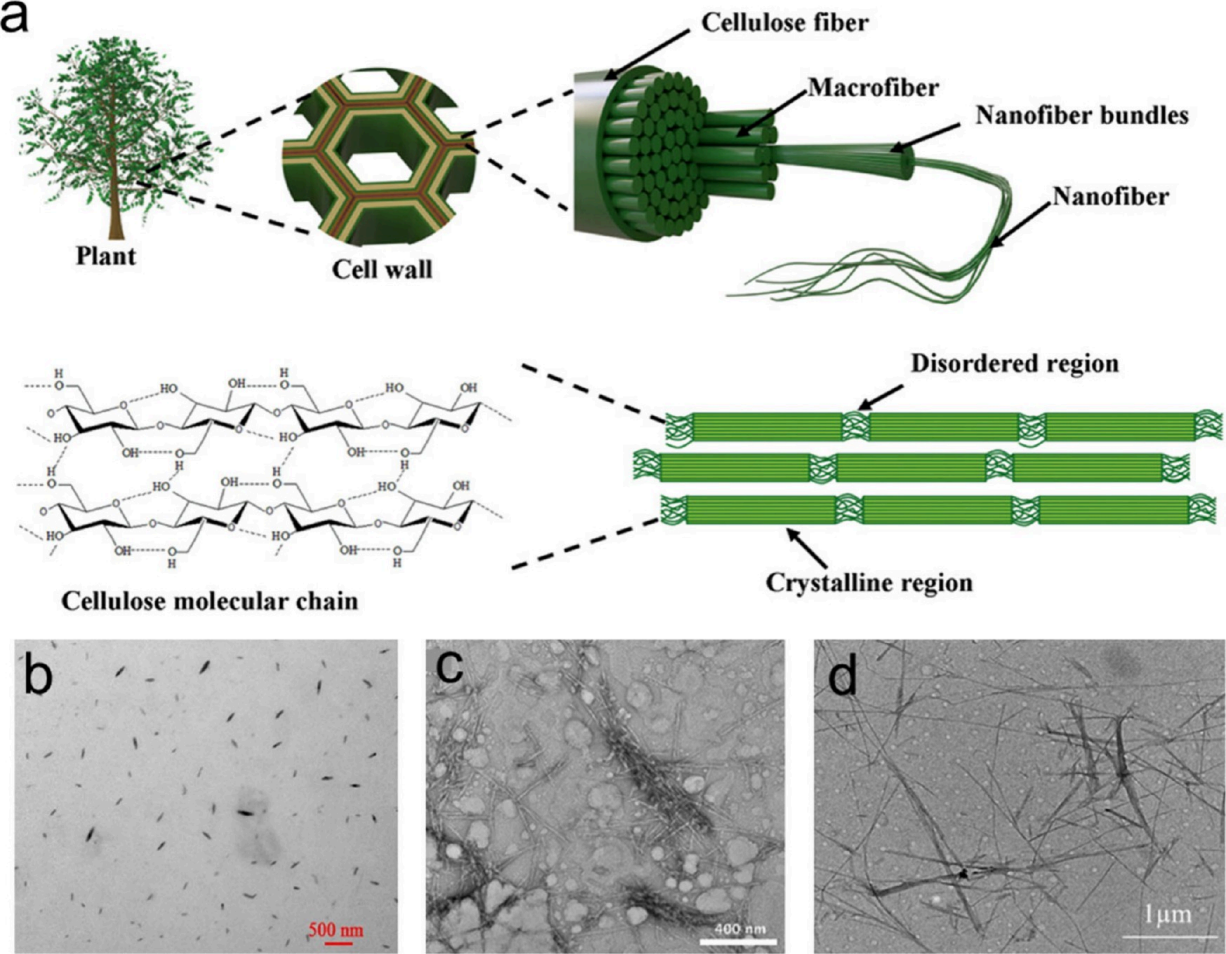
a) Schematic diagram showing the isolation of CNCs from plants. Reprinted with permission from ref (33). Copyright 2021 Wiley-VCH. TEM images of CNCs extracted from b) bacterial cellulose, c) wood pulp, and d) tunicate. b) Reprinted with permission from ref (34). Copyright 2023 Elsevier. c) Reprinted with permission from ref (35). Copyright 2020 American Chemical Society. d) Reprinted with permission from ref (36). Copyright 2024 Elsevier.

Cellulose, a highly functionalizable polymer with many existing industrial applications, occupies a prominent position in abundant organic raw materials due to its availability, biocompatibility, biological degradability, and sustainability.^37^ It is a linear natural polymer composed of d-anhydro-glucose (C_6_H_11_O_5_) repeating units linked by 1,4-β-d-glycosidic linkages at the C1 and C4 positions (Figure 2a). There are three hydroxyl groups on each monomer, which result in the formation of a network of intra- and intermolecular bonds along the cellulose chain.^38,39^ Besides, a network of van der Waals connections is established between the chain layers. The van der Waals interactions and the intermolecular hydrogen bonds between hydroxyl groups and oxygens of adjacent molecules promote the arrangement and stabilization of the cellulose molecules into a highly organized structure through crystalline packing.^33^ It gives rise to structures having a width of 2–20 nm and a length of up to a few micrometers, with crystalline rods along the microfibril axis. These crystalline domains are interspersed with amorphous regions in the fibril structure, where the cellulose molecules cannot be stabilized laterally through H-bonding. Compared with the crystalline parts, the density of the amorphous domains is much lower, which facilitates the hydrogen bond formation with other molecules, for instance, water. Note that for some plant species, these amorphous regions can account for up to 50% of the structure, while in bacterial cellulose and cellulose extracted from some algae the crystalline domains, they account for almost 100% of the fibril. Moreover, these crystalline domains are almost defect-free.^40^

As a result of the hierarchically ordered structures and semicrystalline properties of cellulose, different kinds of nanocellulose including CNCs, also with other designations, e.g., cellulose nanowhiskers (CNWs) and nanocrystalline cellulose (NCC), as well as cellulose nanofibers (CNFs) can be isolated from cellulose by employing suitable strategies.^41,42^ Of note, bacterial cellulose (BC), also termed bacterial nanocellulose or microbial cellulose, is generally produced by aerobic bacteria and has an inherent nanoscale dimension.

Among these cellulose nanomaterials, rod-like or needle-shaped CNCs have attracted significant interest from various research communities due to their distinctive structural characteristics and outstanding physicochemical properties, including nontoxicity, stiffness, low density, optical transparency, adaptable surface chemistry, biocompatibility, biodegradability, and sustainability, just to name a few.^43,44^ Acid hydrolysis utilizing concentrated mineral acids, such as sulfuric acid, phosphoric acid, and hydrochloric acid, is currently the most commonly employed process to extract CNCs.^45−47^ However, some limitations such as relatively high corrosion of equipment, excessive degradation of cellulose, and high water consumption greatly hinder the practical applications of these mineral acid hydrolysis-based processes. Recently, to address these limitations, many other techniques such as organic acid hydrolysis, enzymatic hydrolysis, ionic liquid treatment, subcritical water hydrolysis, oxidation method, mechanical refining, and combined processes have been developed to prepare CNCs.^48−52^ Generally, according to the sources of cellulosic raw materials, e.g., wheat straw, cotton linters, algal cellulose, microcrystalline cellulose, bacterial cellulose, wood pulp, and tunicates, and the isolation approaches employed, CNCs with varying morphologies, dimensions, and crystalline properties could be obtained (Figure 2b–d). The dimensions of CNCs range from 100 nm to several micrometers in length and several to tens of nanometers in width, while the crystallinity of CNCs varies from 50% to 88%.

### 1.2 Introduction to Chitin Nanocrystals (ChNCs)

**Figure 3:**
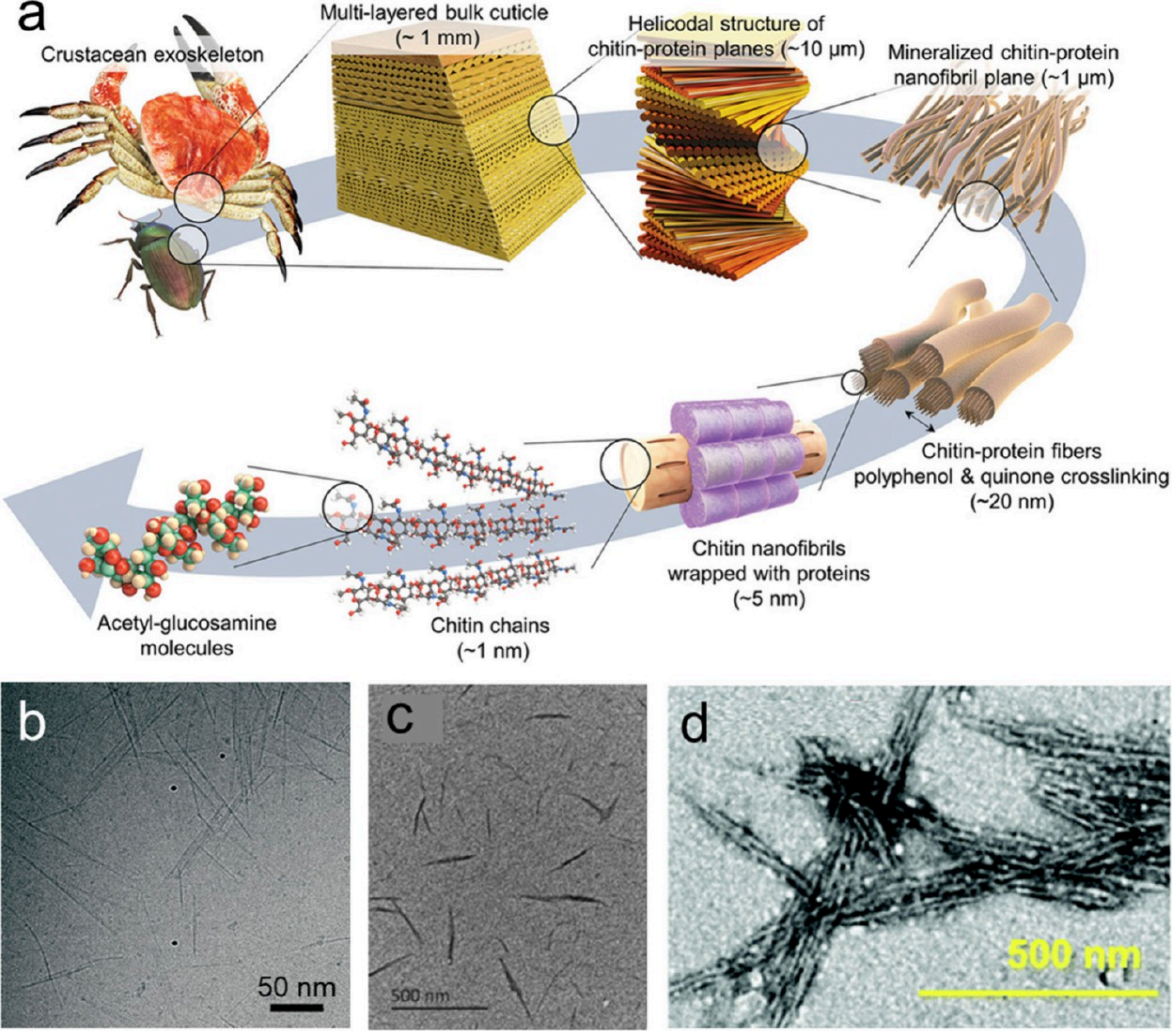
a) Schematic illustration of the hierarchical organization of the exoskeleton of lobster. Reprinted with permission from ref (53). Copyright 2022 Wiley-VCH. b) TEM image of ChNCs extracted from the chitin of crab shell through acid hydrolysis. Reprinted with permission from ref (54). Copyright 2020 American Chemical Society. c) TEM image of ChNCs extracted from the chitin of crab shell through TEMPO-mediated oxidation. Reprinted with permission from ref (55). Copyright 2022 Elsevier. d) TEM image of ChNCs after periodate oxidation from shrimp chitin. Reprinted with permission from ref (56). Copyright 2021 Royal Society of Chemistry.

Chitin, the second most abundant natural polysaccharide following cellulose, is commonly found in the cell walls of yeast and fungi, insect cuticles, shrimp shells, and tendons and shells of lobster and crab, and has the characteristics of biodegradability, biocompatibility, nontoxicity, and antibacterial activity as well as low immunogenicity.^57−59^ In 1811, chitin was extracted from mushrooms by the French botanist Henri Braconnot.^60^ Chitin is a linear polymer comprising repeated N-acetyl-d-glucosamine units linked with β-1,4-glycosidic bonds (Figure 3a), and it is the most prevalent biopolymer containing nitrogen on the planet.^61,62^ Chitin generally exists in nature in the form of ordered, crystalline microfibrils. The crystalline structures of chitin consist of three distinct forms, i.e., α-chitin, β-chitin, and γ-chitin. These structures exhibit variations in the arrangement and packing of chitin molecular chains. In the cuticles of arthropods and fungi, chitin is in the α-form, showing antiparallel structures. β-Chitin with parallel arrangements was mainly extracted from marine fish cartilage. However, γ-chitin is a mixture of α-chitin and β-chitin, characterized by both parallel and antiparallel chain structures.^33,63,64^ Among the three types of crystals, α-chitin is the most prevalent and stable form, with the highest degree of crystallinity and the strongest intermolecular interactions.

ChNCs, the crystalline components isolated from chitin sources at the nanoscale, are also rod-shaped structures with an average length of about 100–1500 nm and a diameter of about 5–80 nm.^61^ Similar to the preparation of CNCs, the primary approach for preparing ChNCs is also acid hydrolysis. During the acid hydrolysis process, the hydrolysis and dissolution of chitin primarily occur in the amorphous regions, resulting in the formation of rod-like ChNCs (Figure 3a).^61,65^Figure 3b shows the ChNCs extracted from crab shell using the acid hydrolysis method. Except for acid hydrolysis, many other approaches, such as 2,2,6,6-tetramethylpiperidine-1-oxyl (TEMPO)-mediated oxidation (Figure 3c),^55^ periodate oxidation (Figure 3d),^56^ ammonium persulfate (APS) oxidation,^66^ and mechanical disintegration,^67^ have also been applied to isolate ChNCs. ChNCs have a high surface area and aspect ratio as well as low density while retaining the original chitin properties. Until now, ChNCs have been widely utilized in water purification, packaging products, cosmetics, food industry, biomedicine electronics, and other fields.

### 1.3 CNCs-Based Chiral Nematic Structures in Nature

**Figure 4:**
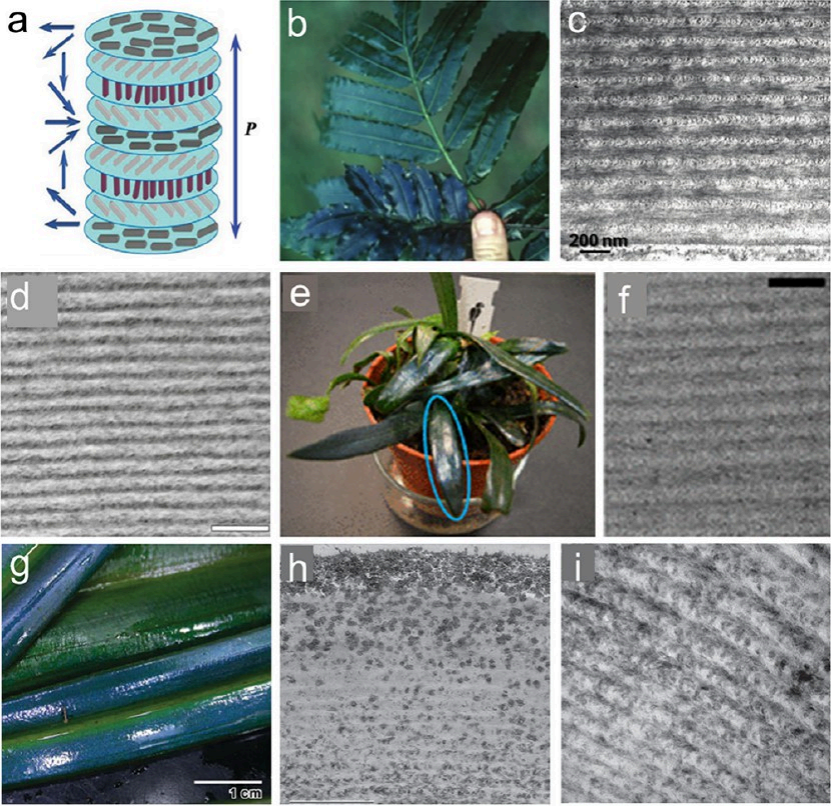
a) Helicoidal arrangement of molecules in chiral nematic liquid crystals. Reprinted with permission from ref (68). Copyright 2018 Wiley-VCH. b) Photograph of *Danaea nodosa*. Reprinted with permission from ref (69). Copyright 2007 University of Chicago Press. c) Cross-sectional TEM image of the cell wall of a juvenile *Danaea nodosa* leaf. Reprinted with permission from ref (70). Copyright 1993 Wiley-VCH. d) TEM image of helicoidal cell wall of *Elaphoglossum herminieri*. Reprinted with permission under a Creative Commons Attribution (CC BY) License from ref (71). Copyright 2024 Oxford University Press. e) Photograph of *Microsorum thailandicum*. f) TEM image of the adaxial cell wall. Reprinted with permission from ref (72). Copyright 2018 Royal Society. g) Photograph of *Mapania caudata*. h) TEM image of the adaxial wall near the surface. i) TEM image of the central part of the adaxial wall. Reprinted with permission from ref (73). Copyright 2013 Oxford University Press.

Chiral nematic structure, also referred to as a helicoidal structure, has chirality at the molecular level and a nonsuperimposable helicoidal superstructure at the macroscale level.^68^ As shown in Figure 4a, the building blocks of the chiral nematic structure exhibit spatial helical orientation along the helical axis. Since the helical direction of the director cannot be superimposed with its mirror image, this structure becomes chiral. The arrangement of helicoidal building blocks is usually characterized by two parameters, i.e., twist handedness and helical pitch (P).^74^ The handedness characterizes how the direction of the molecules rotate along the helical axis, which can be clockwise (referred to as left-handed) or counterclockwise (referred to as right-handed). Helical pitch is the distance over which the director of chiral nematic liquid crystalline rotates a full 360°.^75^

Naturally occurring cellulosic chiral nematic architecture has been discovered in the cell walls of a variety of land plants including ferns, mosses, angiosperms, and gymnosperms.^71,76,77^ The interesting helicoidal structures resulted in vivid structural colors as found in many plants and insects. A typical example is fern *Danaea nodosa*, which displays a strong blue iridescent color in its young leaves (Figure 4b). The strong blue iridescent color is caused by the constructive interference of the thin films composed of multilayers of cellulose microfibrils in adaxial epidermal cell walls (Figure 4c).^70^ The brilliant structural color has also been observed in the leaves of many other fern species, such as *Elaphoglossum herminieri* and *Microsorum thailandicum*, which possess a similar helicoidal ordering of chiral nematic structures (Figure 4d). Iridescence in the ferns *Elaphoglossum herminieri* and *Microsorum thailandicum* is caused by the multilayered structure of their outermost cell walls, where the cellulose microfibrils of each layer are arranged in slightly different directions, resulting in helical patterns of cellulose direction by the stacked layers (Figure 4e–f).^72^

The *Mapania caudata*, showing a blue-green iridescent color, provides a different example of iridescent chiral nematic cellulose containing silica nanoparticles (Figure 4g).^73^ Silica nanoparticles spread within the graded-pitch chiral nematic organization of microfibrils (Figure 4h, i) and contribute to the blue iridescence of the entire structure. Of note, the blue color disappears after the removal of silica from the walls.

**Figure 5:**
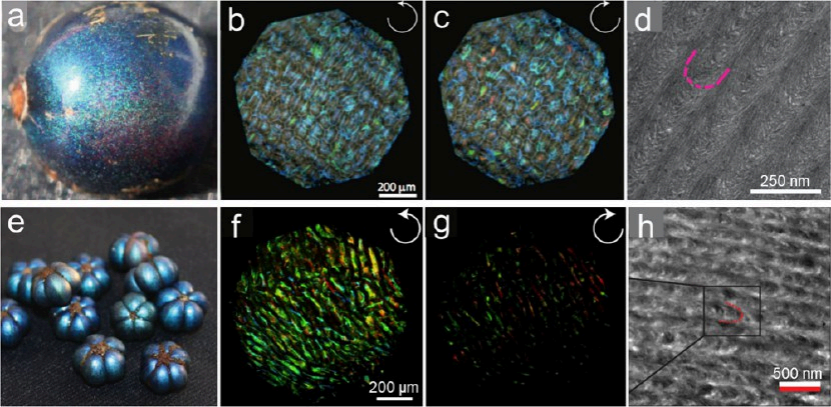
a) Picture of a *Pollia condensata* fruit. b,c) Microscope image in the two circular polarization channels showing the strongly pointillistic coloration originating in individually colored cells of the epicarp. d) TEM image of the epicarp showing the typical helicoidal motif (red line shows one pitch). Reprinted with permission from ref (78). Copyright 2012 National Academy of Sciences. e) Picture of *Margaritaria nobilis* fruits. f,g) Microscope image of a fresh fruit in different circular polarization configurations. h) TEM image of the cell wall of *Margaritaria nobilis* fruits. Reprinted with permission under a Creative Commons Attribution 4.0 International License from ref (79). Copyright 2016, The Royal Society.

The chiral nematic structure that forms the iridescent effect does not only exist in leaves but also in fruits. Generally, various pigments, e.g., flavonoids, carotenoids, and betalains, are responsible for the colors of fruits. However, the fruits of some plant species, such as *Pollia condensata* and *Margaritaria nobilis*, are able to show highly metallic and intense color via nanostructured multilayered cell walls. *Pollia condensata* (Figure 5a) is a kind of African forest understory species showing a shiny metallic blue color, which is mainly due to the chiral nematic structures present in the cell walls of the epicarp cells. In dried fruits, this structure can be preserved for many years.^78,80^ Moreover, the cellulose microfibrils in each cell are arranged in a clockwise or counterclockwise helical orientation, allowing each cell to randomly produce left-handed or right-handed circularly polarized light (CPL) (Figure 5b and c). Figure 5d shows the multilayered helicoidal structure comprising numerous twisted arcs, which are responsible for fruit coloration. The fruits of *Margaritaria nobilis* also exhibit a metallic greenish-blue color (Figure 5e). The fresh fruits show strong circularly polarized reflections similar to those of *Pollia condensata* fruit. However, unlike the left- and right-handed circular polarizations detected in *Pollia condensata*, only left-handed polarization is reflected in *Margaritaria nobilis* (Figure 5f and g). Using TEM and polarization-resolved spectroscopy, Vignolini et al. found that the intense greenish-blue coloration is caused by the helical structures assembled by the cellulose in the multilayered cell walls (Figure 5h).^79^ Additionally, the structural color of the *Margaritaria nobilis* fruit disappears when it is dry. This is mainly due to the shrinkage of the seeds, which generates an air layer between the seeds and the fruit, reducing the saturation and contrast of the structural color.

### 1.4 Chitin-Based Chiral Nematic Structures in Nature

Structural color and iridescence are not limited to plants but are also commonly observed in the animal kingdom. Note that chitin macromolecules, as the basic building blocks of many arthropods, e.g., insects, crustaceans, and spiders, can also self-assemble into helicoidal structures and generate chiral nematic liquid crystal phases due to their chiral nature. As a Bragg medium, the chiral nematic phase exhibits the ability to selectively reflect light, and the helical pitch of the chiral nematic phase determines the wavelength of reflection.^82^ The detailed self-assembly mechanism will be discussed in the next section.

**Figure 6:**
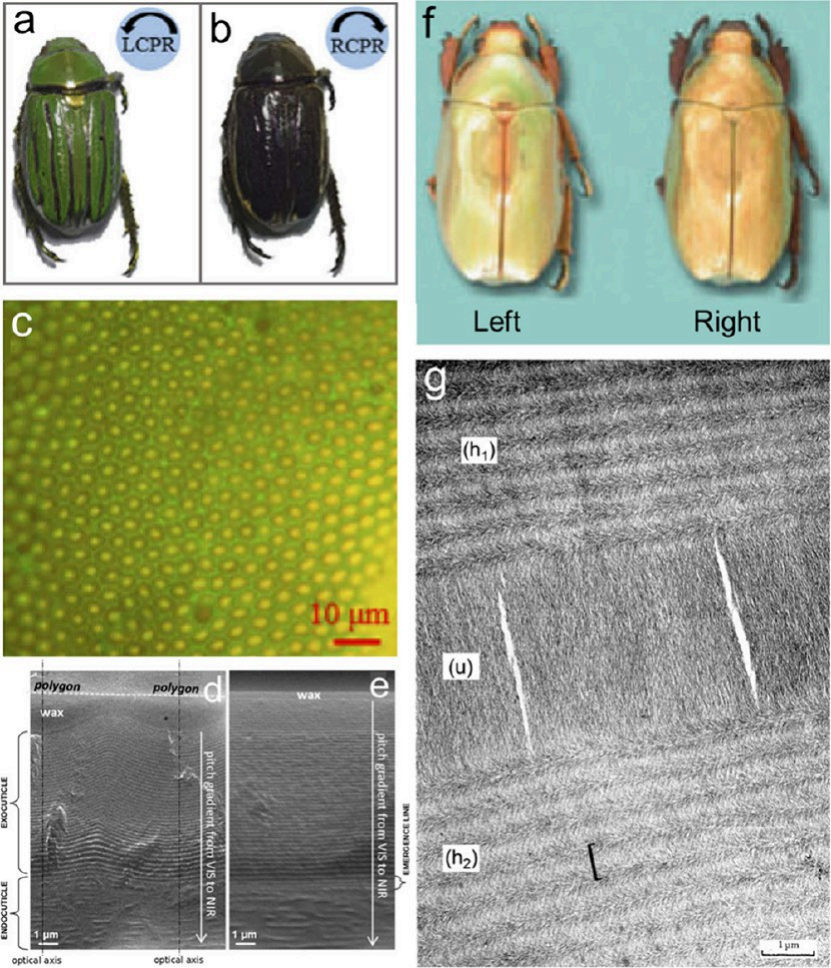
a) Photograph of the beetle *Chrysina gloriosa* under a left circular polarizer. b) Photograph of the beetle *Chrysina gloriosa* under a right circular polarizer. c) A microscopy image shows the exoskeleton of beetle *Chrysina gloriosa*. Reprinted with permission from ref (81). Copyright 2021 Optica Publishing Group. d) Cross-sectional SEM image of green stripes. e) Cross-sectional SEM image of silver stripes. Reprinted with permission from ref (82). Copyright 2017 Elsevier. f) Photograph of *Plusiotis resplendens*. Reprinted with permission from ref (83). Copyright 2005 Springer Nature. g) Electron micrograph showing a cross-section of the reflective layer of *Plusiotis resplendens*. Reprinted with permission from ref (84). Copyright 1971 The Royal Society.

*Chrysina gloriosa* shows a brilliant metallic appearance under a left circular polarizer, while the bright green reflection of the beetle will disappear under a right circular polarizer (Figure 6a and b).^81^ This is mainly due to the selective reflection of the left CPL by the exocuticle of the beetle *Chrysina gloriosa*. As shown in Figure 6c, the green stripe exhibits an exoskeleton consisting of hexagonal cells (with an average diameter of about 10 μm), which contain bright yellow cores embedded within green cells with a yellow border. The typical chiral nematic fingerprint texture can be clearly recognized from the SEM images of the *Chrysina gloriosa* cuticle (Figure 6d and e).^82^

Apart from *Plusiotis resplendens*, in all examined Plusiotis species, the reflected light is entirely left circularly polarized across the visible spectrum. However, *Plusiotis resplendens* reflects not only left CPL but also right CPL (Figure 6f),^83^ which is mainly because of more complex helicoidal structures present in the cuticle of Plusiotis resplendens. As shown in Figure 6g, the cuticles exhibit a three-layered structure in which half-wave retardation plates are located in two left-handed helicoids.^84^ When light passes through this three-layered structure, the left-handed CPL is reflected via the upper left-handed helicoids. However, the right-handed CPL will traverse half-wave retardation plates and be converted to left-handed, which will further interact with the left-handed helicoids at the bottom and then be reflected as a right-handed light after traversing a half-wave retardation plate. Thus, both left- and right-handed CPL can be successfully reflected with the same spectral content.

## 2 SELF-ASSEMBLY OF CNCs AND ChNCs

**Figure 7:**
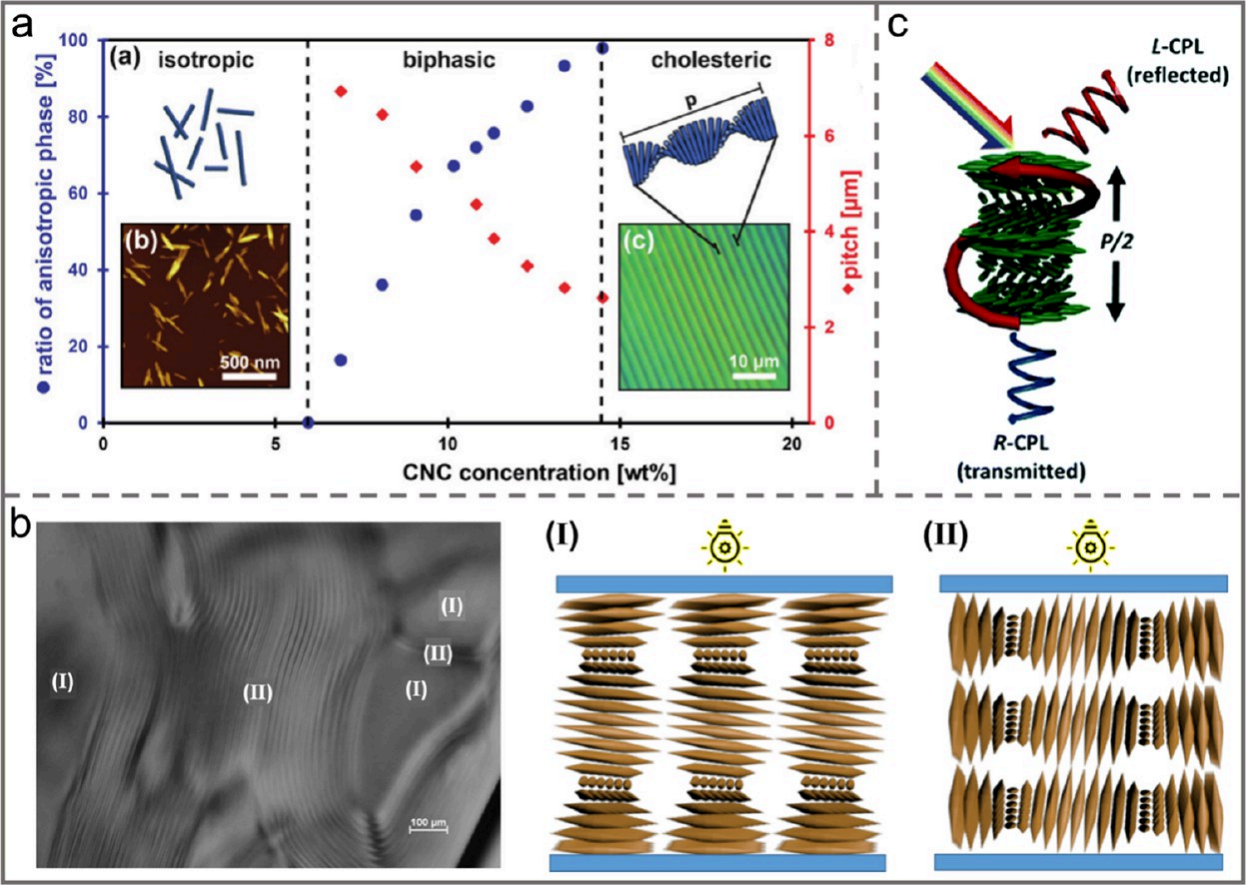
a) Phase diagram illustrating the transition of isotropic-to-chiral nematic phase and the corresponding equilibrium pitch with the increasing of CNC concentration. Reprinted with permission under a Creative Commons Attribution (CC BY) License from ref (18). Copyright 2017 Wiley-VCH. b) Regions show the (I) planar texture and (II) fingerprint texture. Reprinted with permission under a Creative Commons Attribution (CC BY) License from ref (85). Copyright 2015 MDPI. c) Schematic diagram showing the chiral nematic structures formed by CNCs that can selectively reflect CPL. Reprinted with permission from ref (86). Copyright 2019 Royal Society of Chemistry.

The birefringent property of CNC suspension was reported by Marchessault and co-workers in 1959.^87^ However, the chiral nematic (cholesteric) phase of CNC suspension was not identified until 1992 by Gray et al.^88^ Subsequently, Gray and co-workers found that the chiral nematic structure of CNC suspensions can be preserved in thin films after water evaporation.^89,90^ As shown in Figure 7a, only when the concentration of CNC is higher than a critical point does an ordered chiral nematic phase appear, forming an orientationally anisotropic phase.^18^ In the dilute regime (isotropic phase) with the CNC concentration below around 6 wt %, these nanocrystals are randomly dispersed and oriented so that the suspension appears as a single isotropic phase. When the concentration of CNC exceeds the critical threshold of around 6 wt %, a second phase is observed, resulting in the coexistence of an anisotropic phase in the lower layer and an isotropic phase in the upper layer. Note that the volume of the anisotropic phase increases as the suspension concentration increases. As the concentration of CNC increases further to 14.5 wt %, the entire suspension becomes anisotropic. The liquid crystalline textures formed during the self-assembly process can be detected using a polarized light microscope (PLM). Of note, different textures are determined by the relative orientation between the helicoidal axis and the PLM. As shown in Figure 7b, the planar texture (I) and fingerprint texture (II) of the CNC suspension can be clearly observed.^85^ Birefringence occurs when the helicoidal axes are perpendicular to a substrate, which is known as a planar structure. However, when the helicoidal axes are parallel to a substrate, alternating bright and dark stripes are formed, which are known as a fingerprint structure.

The helix orientation of CNC chiral nematic phase has been demonstrated to be left-handed (Figure 7c).^86^ The reflected wavelength of chiral nematic structures (λ_max_) is usually expressed according to the equation below:^91^
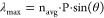
Where P represents the helical pitch of chiral nematic structure, n_avg_ is the average refractive index of the substance, and θ refers to the angle of incident light. When half of the pitch (P/2) matches the wavelength of visible light, the material appears to be iridescent. The light reflected by the chiral nematic structure is circularly polarized, and its handedness is mainly dependent on the chirality of the structure.^86^

Similar to CNCs, ChNCs can also spontaneously self-assemble into chiral nematic phases in an aqueous suspension when the concentration is above a threshold concentration. Furthermore, the chiral nematic structures are retained upon drying the dispersion to produce solid iridescent films.^92−94^ For ChNCs, the wavelength of light reflected by chiral nematic structures can also be described according to the same equation for CNCs. The iridescent colors in ChNC films with chiral nematic structures are also angle-dependent and are determined by the average reflective index and pitch of the helical structures.

## 3 INFLUENCE AND REGULATION OF LIQUID CRYSTAL BEHAVIORS OF CNCs

A CNC suspension generates a film with an iridescent color at visible wavelength after complete evaporation of water. Various crucial parameters including internal factors (e.g., morphology, dimension, and surface charge density) and external factors (electrical field, magnetic field, and mechanical stress) could be tuned to realize the adjustment of the pitch of the chiral nematic structures in CNC suspensions, thus leading to the formation of CNC films with desired structural colors.

### 3.1 Effects of Internal Factors During the Self-Assembly Process

As reported in many previous studies, the size of CNC particles has a significant impact on the evolution of the self-assembly process and the optical properties of CNC films. Thus, controlling the size of CNCs has been actively pursued to obtain self-assembled CNC films with the desired chiral nematic structures. He and co-workers investigated the self-assembly behaviors of carboxylated tunicate CNCs (tCNCs), prepared through ammonium persulfate (APS) oxidation in combination with subsequent ultrasonication.^95^ They found that tCNCs with smaller sizes require a slightly higher critical concentration to form the anisotropic phase. Two different CNCs with similar diameters, zeta potential, and sulfur group contents but different lengths were isolated from softwood by sulfuric acid hydrolysis.^96^ To understand the interaction and self-assembly of CNCs with different lengths in an aqueous suspension, experimental small-angle X-ray scattering (SAXS) and the theoretical Derjaguin–Landau–Verwey–Overbeek (DLVO) theory were applied. The results showed that the balance between attractive and repulsive interactions has a significant influence on the chiral nematic self-assembly of CNCs with different morphologies. Zhao et al. fabricated CNCs with various aspect ratios through changing the temperature of hydrolysis and acid-to-pulp ratio.^97^ CNCs with higher aspect ratios obtained at lower hydrolysis temperatures and acid-to-pulp ratios are more likely to form chiral nematic structures and generate films with structural colors. The obtained film reflected natural light in wavelengths ranging from 360 to 695 nm and exhibited structural colors from blue to orange. It is noteworthy that strong acid hydrolysis would disrupt the cellulose lattice planes, which is not conducive to the formation of chiral nematic structures. Generally, unlike rod-like particles, spherical particle suspensions with a concentration higher than the critical value tend to form crystalline colloids instead of liquid crystalline phases. However, Wang and co-workers reported that highly polydisperse spherical CNCs can also form a liquid crystalline phase in suspension.^98^ Spherical CNCs started to show a liquid crystalline phase when the concentration of CNC exceeded 3.9 wt %. When the concentration increased to 4.5 wt %, the suspension displayed a chromatic color and had a banded texture. With a further increase in CNC concentration (7.1 wt %), a crosshatch pattern was detected. Vermal et al. extracted CNCs with varying morphologies such as sphere, flake, and needle from different sources.^99^ They found that these CNCs with varying morphologies have different liquid crystalline properties and self-assembly patterns. Only the needle-shaped nanocrystals captured the characteristic fingerprint pattern. A banded pattern was observed for the spherical CNCs, which may be due to the inevitable aggregation of nanocrystals, leading to the change in the effective particle shape of nanocrystals. Nanocrystals with a flakelike morphology could not produce long-range order, thus resulting in the formation of inhomogeneously ordered patterns.

In addition to size and morphology, the surface charge density also strongly influences the self-assembly of CNCs. It was found to affect not only the colloid stability of the CNC suspension but also the chiral nematic pitch of the dry film. Various approaches, including physical adsorption of surfactants, chemical grafting with polymer chains, and changing the synthesis conditions of CNCs, have been developed to tune the surface charge of CNCs. For instance, CNCs with almost identical dimensions but different surface charges were extracted from bleached softwood kraft pulp by using the sulfuric acid hydrolysis method.^100^ It was found that the critical concentration of CNC suspension required to form a chiral nematic phase increased as the surface charge increased. Moreover, there was a surface charge density threshold of around 0.3% S, below or close to which the end-to-end aggregated CNCs tended to form gelation and disturbed the formation of the ordered phase. CNCs with different sulfate contents were fabricated by Yao et al. by changing the sulfuric acid hydrolysis time.^6^ With an increasing hydrolysis time, the sulfate content of the CNC increased, while the size of the CNC decreased. The electrostatic repulsion increased with increasing surface charge density, which resulted in the predominant reflectance peak of the dried CNC films increasing from 271 ± 10 to 517 ± 8 nm. Compared with H_2_SO_4_-hydrolyzed CNCs, the negatively charged carboxylated CNCs obtained by pre-^101^ or post-treatment^102^ with TEMPO can form a chiral nematic phase with apparent optical birefringence at a critical concentration of about 4.1 wt %, which was lower than that of CNCs prepared with H_2_SO_4_ (4.8 wt %). When the concentration increased to 9.0 wt %, the fingerprint could be clearly observed under a PLM, and the helical pitch was about 6 μm.^103^ Fan et al. prepared carboxylated CNCs from bleached cotton pulp via hydrochloric acid hydrolysis in combination with subsequent TEMPO-mediated oxidation.^104^ For CNCs with similar aspect ratios, a higher surface charge density resulted in enhanced dispersity of the negatively charged nanoparticles and the stability of the suspension. When the surface charge density of CNCs was 0.16, 0.56, and 1.00 e·nm^–2^, the CNC suspensions showed a birefringent pattern without clear fingerprint texture. However, clear fingerprint textures can be found when the surface charge density of CNCs increases to 1.25 and 1.42 e·nm^–2^. Lin et al. reported a facile method to prepare carboxylated CNCs from degreasing cotton using aqueous recyclable oxalic acid as the sole catalyst for esterification and hydrolysis.^105^ It was found that the self-assembly behaviors of the CNC were highly determined by the aspect ratio and surface charge. The formation of chiral liquid crystal phases was observed in samples with aspect ratios greater than 5.5 and surface charges higher than 30.0 mV.

Many groups investigated the effect of polymer-graft modification on the CNC self-assembly process. Araki and co-workers produced sterically stabilized CNC suspensions via grafting polyethylene glycol (PEG) onto the surface of the nanocrystals through a carboxylation-amidation procedure.^102^ Owing to the fact that the PEG used in this study was too short to produce steric barriers that shield the surface charge effects, the PEG-grafted CNCs displayed self-assembly behavior similar to the unmodified CNCs. By using the classical surface-initiated atom transfer radical polymerization (SI-ATRP) method, Risteen et al. produced thermally switchable liquid crystalline CNC by regioselective grafting of thermoresponsive polymer poly(*N*-isopropylacrylamide) (PNIPAM) from the reducing ends of the nanocrystals.^106^ They found that the phase transition from liquid crystalline to isotropic was reversible and driven by the collapse of PNIPAM chains on top of the lower critical solution temperature (LCST), leading to a decrease in rod packing density and an increase in translational and rotational degrees of freedom. CNCs surface grafted with poly(PMMAZO) were prepared via ATRP.^107^ The PMMAZO-grafted CNCs displayed smectic-to-nematic transition and nematic-to-isotropic transition at 95 and 135 °C, respectively. When the temperature exceeded 135 °C, the grafted CNC showed lyotropic liquid-crystalline phase behavior and a lyotropic nematic phase in chlorobenzene when the concentration was above 5.1 wt %. With the same method of ATRP, it is also possible to graft poly(styrene) (PSt) on the surface of CNCs.^108^ The PSt-grafted CNCs showed chiral nematic structures in both the thermotropic and lyotropic states. Delepierre et al. reported that end-grafted CNCs could self-assemble into a chiral nematic liquid crystalline phase.^109^ One end of the reducing end-groups of CNC-I and both ends of CNC-II were grafted with poly[2-(2-(2-methoxyethoxy)ethoxy)ethyl acrylate] (POEG_3_A). Compared to the unmodified CNC, the thermal properties and liquid crystalline phase behavior of the CNCs changed after modification. Both modified CNC-I and CNC-II produced chiral nematic tactoids in aqueous suspensions, but the domain sizes and pitches were different. Chiral nematic tactoids could only be formed when the concentration of modified CNC-II exceeded 14 wt %, while a concentration of CNC-I of 10% was sufficient. However, the symmetrical grafting of POEG_3_A onto the reducing end group of CNC-II significantly shortened the time required for the chiral nematic texture formation. CNCs with surface-grafted ionic liquids (CNC-IL) were synthesized by Qin et al.^110^ It was found that positively charged CNC-IL formed a chiral nematic phase at a significantly lower concentration than nonfunctionalized CNCs. In addition, the chiral nematic pitch increased with increasing concentration of CNC-IL due to the increased particle size of the CNCs and the high viscosity of the CNC-IL suspension after surface modification with ILs.

### 3.2 Effects of External Factors During the Self-Assembly Process

The concentration of CNCs, as one of the most easily adjustable external factors, is greatly associated with the microstructure of the chiral nematic phase formed by CNC. Momeni et al. discovered that the chiral nematic pitch decreased with increasing the concentration of CNC.^111^ This is consistent with the results of previous works, where an increase in the coexisting concentration of isotropic and anisotropic phases but a decrease in the chiral nematic pitch of the anisotropic phase was observed as the total concentration of the CNC suspension increased. Two main mechanisms have been used to explain the decrease in pitch as the CNC concentration increases. First, the strength of the total force for twisting increases with increasing CNC content in the suspension, which facilitates the formation of a chiral nematic structure. Second, the ionic strength also increases with increasing concentration of charged CNCs, which reduces the range of exponentially decaying electrostatic repulsion, resulting in a tight alignment of CNCs.^112^ Klockars et al. fabricated CNC films with structural color by casting CNC suspensions with different concentrations of 3, 4, 5, and 6 wt %, and their morphology and optical properties were characterized.^113^ Results showed that the drying time decreased with an increase of concentration. A more dilute CNC suspension required a longer evaporation time, resulting in a film with larger and more uniform crystalline domains, while a more concentrated CNC suspension required a shorter evaporation time, producing a film with smaller domains. This is mainly because the sample assembled from a higher concentration of the suspension reached the kinetic stagnation or gelation stage more quickly after evaporation, and there was not enough time to rearrange into long-range orders. The influence of concentration on the self-assembly behavior of carboxylated CNCs was investigated by Lin et al.^105^ The study found that the suspension was optically isotropic when the concentration was lower than 1.0%. Increasing the concentration of the suspension to 1.0% produced a biphasic suspension with an upper isotropic phase and a bottom anisotropic phase. The suspension became fully anisotropic when the concentration was increased to 3.0%.

Ionic strength is also an essential external factor influencing the self-assembly of CNC into a chiral nematic phase in suspension due to the charged character of CNCs. Generally, the chiral nematic pitch can be controlled by adjusting the ionic strength of the suspension, resulting in the formation of CNC films with tunable reflected colors. Bukharina et al. investigated the effect of ionic strength on the chiral nematic phase of sulfuric acid hydrolyzed CNCs extracted from wood pulp.^114^ Prior to evaporation, an appropriate amount of the NaOH solution was added to the CNC suspension. It was found that the pitch decreased with increasing NaOH concentrations. This was mainly due to the strong interaction between Na^+^ and the surface of CNCs shielding the surface charges and reducing the effect of Coulombic repulsion. Such a phenomenon was also revealed by Lin and coauthors.^115^ Cao and coauthors studied the effect of counterions on the aggregation kinetics and surface charging properties of CNCs in aqueous suspensions containing various inorganic salts.^116^ They found that for monovalent salts, the critical coagulation concentration (CCC) followed the order of Cs^+^ < K^+^ < Na^+^ < Li^+^, consistent with the Hofmeister series, suggesting specific interactions between cations and the CNCs surface. The CCC values for the divalent salts followed the order Mg^2+^ > Ca^2+^ > Ba^2+^, which was the opposite order of counterion ion size. The properties of the counterions also affect the stability of the suspension.

The solvent of CNC suspensions also greatly affects their self-assembly behavior. Of note, CNCs prefer to form aggregates in an apolar solvent owing to strong hydrogen bonds and weak electrostatic repulsion. Thus, so far, the dispersion and self-assembly properties of CNCs are limited to aqueous suspensions or a few organic solvents with high dielectric constants, such as N,N-dimethylformamide (DMF), ethylene glycol, and dimethyl sulfoxide (DMSO).^117−119^ To understand how different solvents affect the self-assembly of CNCs, Bruckner et al. dispersed CNCs in different polar but nonaqueous solvents, such as formamide, *N*-methylformamide (NMF), and DMF.^120^ They developed an aggregation-free method for exchanging solvents in the CNC suspensions. The desired solvent was first mixed with the aqueous suspension of CNCs, and then water was removed through distillation under reduced pressure. The solvent with a high dielectric permittivity was found to have the ability to significantly accelerate the self-assembly process and reduce the concentration dependence of the helical pitch. High-permittivity solvents increased the degree of order in CNC suspensions, resulting in a shorter pitch and lower threshold for liquid crystallinity. In contrast, low-permittivity solvents hindered the formation of helical superstructures due to kinetic arrest. The electrostatic repulsion is a key factor for CNC to form chiral nematic phases in a suspension. However, some researchers reported that the modified CNCs are also capable of forming a chiral nematic phase, suggesting that surface charge is not a prerequisite for isotropic self-assembly.^108,121^ Saraiva et al. reported the production of hydrophobic CNCs through surfactant-functionalization of sulfuric acid hydrolyzed CNCs with a commercial surfactant of STEPFAC 8170-U.^122^ Surfactant-functionalized CNCs suspended in toluene formed the chiral nematic phase within a few hours, which was unexpectedly fast compared with pure CNCs in water. Furthermore, the concentration of the suspensions could reach up to 50 wt %. They also found that the pitch values of the chiral nematic phase decreased with an increase in CNC concentration. In addition to surface modification, CNCs can be dispersed in toluene by adding surfactants to the suspension. In the study reported by Frka-Petesic et al., CNC toluene suspensions were obtained by adding Beycostat NA surfactant (BNA) at a surfactant/CNC ratio of 4/1 (w/w) to aqueous CNC dispersions.^123^ CNCs in toluene can spontaneously form iridescent liquid crystal phases. The cholesteric orientation and the periodicity could be controlled by using electric fields to achieve uniformity of macroscopic samples and dynamic adjustment of the iridescent hues.

**Figure 8:**
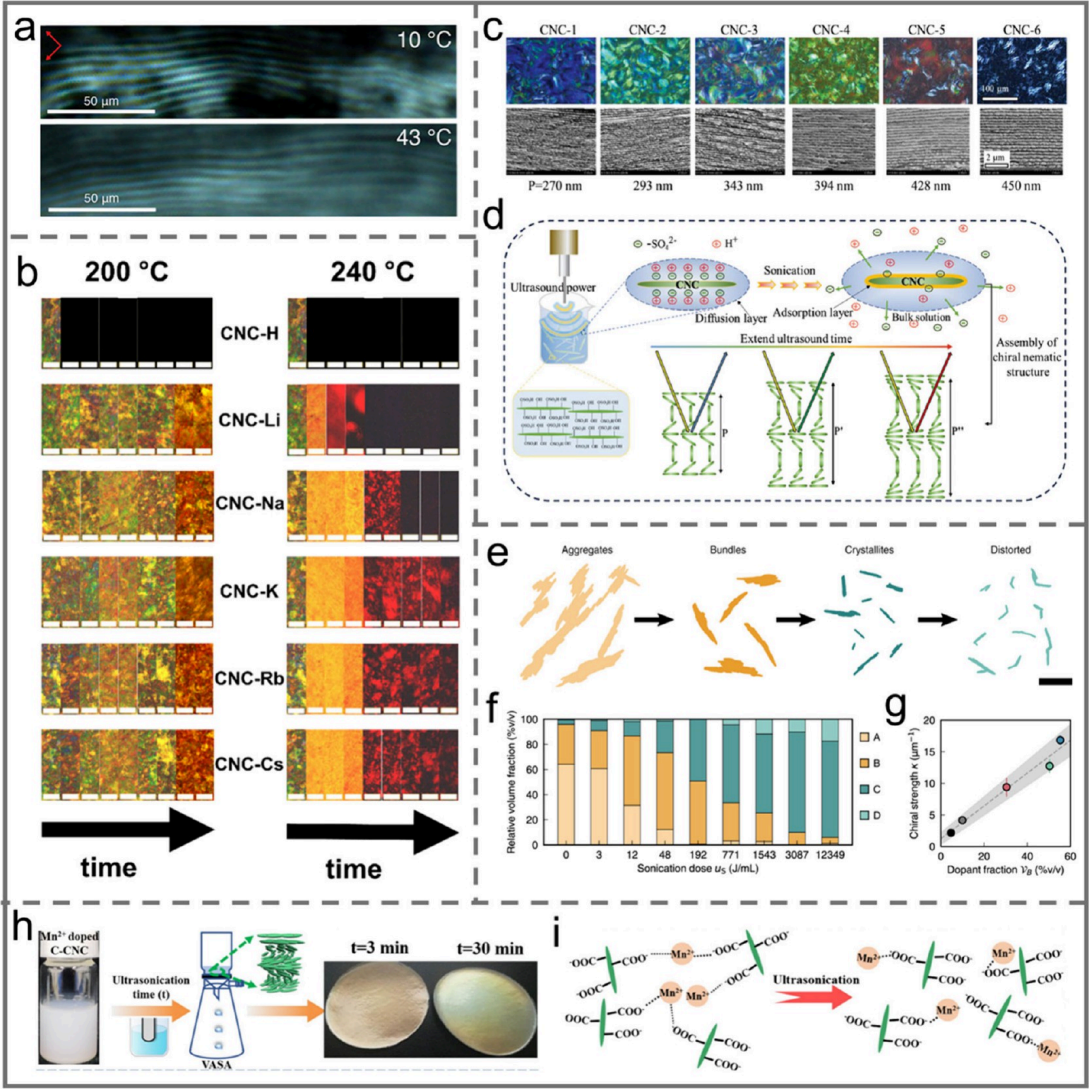
a) PLM images of the bulk chiral nematic phase at 10 and 43 °C. Reprinted with permission from ref (20). Copyright 2018 American Chemical Society. b) PLM images of CNC-X films under different annealing temperatures and annealing times. The annealing times from left to right in each group of images are 0 h, 30 min, 1, 3, 6, 12, 18, 24, and 48 h. Scale bar: 100 μm. Reprinted with permission from ref (124). Copyright 2021 Elsevier. c) PLM images and cross-sectional SEM images of CNC iridescent films with different ultrasonic time. d) Schematic illustration of the mechanism of sonication-driven increase in pitch. Reprinted with permission from ref (125). Copyright 2024 Wiley-VCH. e) Schematic illustration of the evolution of particle distribution under sonication. Scale bar 200 nm. f) The relationship between relative volume fraction of each particle subpopulation and sonication dose. g) Modulation of chiral strength with relative dopant volume fraction. Reprinted with permission under a Creative Commons Attribution (CC BY) License from ref (29). Copyright 2022 Springer Nature. h) Schematic illustration of the fabrication of Mn^2+^ doped C–CNC iridescent films and images of the color of the prepared iridescent films as a function of ultrasound time. i) Schematic diagram of the mechanism of ultrasonic destruction of C–CNC agglomerates. Reprinted with permission from ref (126). Copyright 2022 Elsevier.

External physical methods can be used to tune the self-assembly of CNC during the fabrication of free-standing structurally colored CNC films. Evaporation-induced self-assembly (EISA) is the most commonly used method to produce colored CNC films. The evaporation process can be altered via simply changing the environment of the suspension. For instance, inputting external energy through heating was reported for constructing structural color films with CNC prepared by the sulfuric acid hydrolysis method. The surface charges of sulfuric-acid-treated CNCs are highly sensitive to thermal treatment as the elevated temperature causes the desulfation of sulfate ester groups on CNC surfaces.^32^ These functional groups play a key role in the formation of long-range ordered chiral nematic structures. Vanderfleet et al. investigated the influence of hydrothermal treatments on the properties such as the uniformity, colloidal stability, and color of the sulfated, phosphate, and carboxylated CNC suspensions.^127^ It was found that the morphology, molecular weight, and crystallinity of CNCs were almost unchanged after treatment, while the surface charge content decreased significantly, resulting in the loss of colloidal stability and aggregation of CNCs. Compared with phosphated and carboxylated CNCs, sulfated CNCs were most likely to decrease colloidal stability and form CNC aggregates. The study reported by Nyström et al. demonstrated that the pitch of the chiral nematic phase formed by carboxylated CNCs was not affected by temperature changes between 10 and 43 °C (Figure 8a).^20^ Liu et al. found that a free-standing film with perfect chiral nematic liquid crystal characteristics can be obtained at a low temperature of 30 °C.^128^ As the casting temperature increased, the liquid crystal structures of the CNC films underwent a process of gradually changing from chiral nematic phases to nematic phases until no chiral nematic phase remained due to the deep desulfurization of CNCs. Beck and coauthors prepared solid iridescent CNC films with predefined patterns by heating aqueous CNC suspensions differentially during film casting.^129^ It was found that the thickness and chiral nematic pitches of the patterned areas were different from those of the surrounding films. The red-shifted pattern can be formed at a higher casting temperature, while the blue-shifted pattern was obtained by decreasing the casting temperature. Moreover, the red-shifted area was thicker than the blue-shifted area due to the longer chiral nematic pitch. Tran and coauthors explained the red-shift phenomenon detected in CNC film when external energy was input by heating. This may be due to the kinetic entrapment of water molecules between the layers of CNC rod resulting in the increase of helical pitch value, while also introducing obvious disorders into the produced chiral nematic structures.^16^ Applying heat treatment during film preparation affects the self-assembly of CNCs, while heat treatment of the dried CNC film can also have an effect on the film. D’Acierno et al. reported the changes in the optical and structural properties of the iridescent CNC films with different monovalent cations (CNC-H, CNC-Li, CNC-Na, CNC-K, CNC-Rb, CNC-Cs) after thermal annealing at 200 and 240 °C for up to 2 days.^124^ They found that the birefringent properties of the CNC-H films were destroyed within a few seconds after annealing at 200 °C, along with the rapid disappearance of the domain structure, indicating poor thermal stability (Figure 8b). For CNC-Li, CNC-Na, CNC-K, CNC-Rb, and CNC-Cs films, the chiral nematic structures were retained in these films due to the structural and chemical stability of the cellulose backbone and the large surface alkali ions. Furthermore, the stability of CNC-X films increased as the radius of the alkali ions increased. Guidetti et al. also investigated the impact of thermal treatments on the optical properties of chiral nematic CNC films.^130^ The research demonstrated that the helicoidal architecture and chiral optical response of CNC films can be maintained at temperatures up to 250 °C through base treatment and cross-linking with glutaraldehyde. Additionally, exposure to a vacuum allows preservation of the helicoidal arrangement up to 900 °C, resulting in the production of aromatic chiral carbon.

Ultrasonic treatment prevents the aggregation of CNCs by destroying the hydrogen bonding, resulting in small CNC particles and stable CNC suspensions.^131,132^ Recently, ultrasonic treatment has been established as an efficient way to tune the chiral nematic pitch of CNCs in suspension by changing the applied energy.^29,125,133^ Of note, it was found that short-term sonication was sufficient to disperse CNCs, whereas long-term sonication was counterproductive as it increased the critical concentration required for the liquid crystalline phase formation.^134^ Moreover, the influence of sonication on the treated CNC suspension is cumulative and permanent. Chen et al. demonstrated that ultrasonic treatment of CNC suspensions before film casting increased the chiral nematic pitch and gave rise to the final iridescent CNC film with reflection bands of longer wavelengths (Figure 8c).^125^ This is mainly due to the fact that some of the incompletely dialyzed counterions from acidolysis were bound to the surface layer of CNCs, partially shielding the surface negative charge of CNCs (Figure 8d). Without ultrasonic treatment, the reduced electrostatic repulsion allows the CNC to self-assemble with a shorter chiral nematic pitch. After ultrasonication, the shielding effect was diminished and the electrostatic repulsion during CNC self-assembly increased due to the gradual input of energy into the system causing some counterions in the surface layer of the CNC to leave and diffuse into the suspension, thus enlarging the pitch of the CNC chiral nematic structure. In contrast to such a model, Parton et al. proposed a chiral dopant model that composite CNCs, namely CNC bundles, functioning as chiral dopants, are the main driving force for the pitch increase upon sonication.^29^ Through investigating the evolution in particle morphology upon sonication, it was found that aggregates and bundles were initially dominant, and upon sonication, aggregates were broken into smaller structures, while the bundles are more persistent and require higher doses to be broken down into individual elementary crystallites, including crystallites and distorted crystallites (Figure 8e and f). Among these four subpopulations, the bundle population was identified as the chiral dopant. Moreover, the chiral strength κ, extrapolated from the measurements of the volume fraction and the pitch, shows a clear positive trend with dopant abundance (Figure 8g). Yang et al. changed the input sonication energy to broaden the color spectrum of photonic oxidized CNC (_OX_CNC) films.^135^ For the _OX_CNCs with low surface charges, no obvious color change was observed, even with a relatively high sonication energy input. By contrast, for _OX_CNCs with high surface charges exhibiting strong electrostatic repulsion, a stronger red color could be observed with an increase in the input sonication energy. Sui et al. reported that the color uniformity of CNC films obtained from suspensions was significantly reduced without ultrasonication due to the competition between the surface energy and nematic energy between CNCs during water evaporation.^136^ The color uniformity improved after applied ultrasonication on the CNC suspension, and the fingerprint structure in the films was clearly observed by PLM. When CNCs are mixed with metal ions to prepare iridescent films, ultrasonication can also be used to improve the dispersibility of the suspension. Qin et al. reported the fabrication of Mn^2+^-doped carboxylated CNC iridescent films through ultrasonication pretreatment followed by the vacuum-assisted self-assembly (VASA) method.^126^ The color of the composite films could be controlled by changing the ultrasonication time (Figure 8h). Ultrasonication treatment not only facilitated the uniform dispersion of CNC in the suspension but also partially destroyed the strong bonding between carboxylate and Mn^2+^ (Figure 8i).

**Figure 9:**
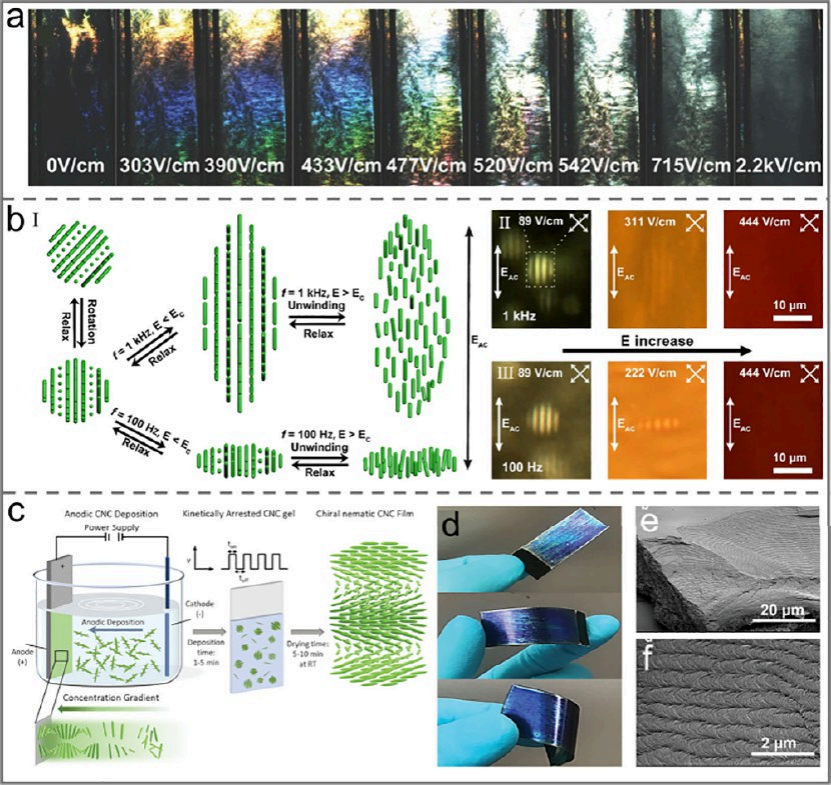
a) Evolution of iridescence in chiral nematic suspension with increasing electric field. Reprinted with permission from ref (123). Copyright 2017 Wiley-VCH. b) Schematic diagram of the reversible electrical response properties of CNC tactoid and PLM images of tactoid with increasing E. Reprinted with permission from ref (137). Copyright 2020, American Chemical Society. c) Schematic diagram of the self-assembly of CNC by electrophoretic deposition. d) Photographs of the electrodeposited CNC film on a flexible substrate. e–f) SEM images of CNC film illustrating the chiral nematic structures under different magnifications. Reprinted with permission from ref (138). Copyright 2022, Wiley-VCH.

External electric fields have also been verified as a facile yet efficient tool to control the self-assembly of CNCs. Bordel et al. reported that CNCs were aligned along the direction of the electric field and the birefringence of the suspension disappeared immediately when the electric field was turned off.^139^ Additionally, the orientation and degree of alignment of the rod-like particles can be controlled by changing the applied field strength and frequency during film formation. This method provides dynamic modulation of the structural colors in addition to sample uniformity at the macroscopic scale and exact pitch adjustment.^123^ At a lower field, the light intensity increased with increasing electric field intensity, accompanied by a red-shift in color. However, as the intensity increased further, the color became a scattering white and finally disappeared (Figure 9a). Qu et al. reported the use of an alternating current electric field to dynamically control the chiral nematic and nematic structures of CNC tactoids.^137^ When a weak electric field was applied, the helical axes of the CNC tactoids were perpendicular to the electric field. This is mainly due to the positive dielectric anisotropy of CNCs and the synergistic effect of assembled CNCs. When the frequency of the applied electric field decreased or the strength increased, the pitch of CNC tactoids increased and then shifted from a chiral nematic structure to a nematic structure. At the same time, the response of CNC tactoids was significantly different at different frequencies (Figure 9b). Atifi et al. successfully fabricated photonic CNC films with long-range chiral nematic order in minutes via electrophoretic deposition (EPD)-induced self-assembly.^138^ When an electric field was applied between the electrodes, CNCs moved toward the anode and generated a concentration gradient, which facilitated CNC interaction and self-assembly (Figure 9c). A relatively homogeneous and dense gel was formed near the deposition electrode after the aggregation of the CNCs. The drying time for mechanically stable gels typically did not exceed 10 min, either in situ or removed for drying. Furthermore, different grades of well-dispersed CNCs could be deposited on rigid and flexible substrates in a very short time by applying the pulsed EPD technique (Figure 9d). The SEM images clearly showed the homogeneous layered structure, which was characteristic of a left-handed helical arrangement for CNCs (Figure 9e and f). The thickness of the films was primarily affected by the nature of CNCs and the applied EPD parameters. Furthermore, the reflection intensity of the obtained films could be adjusted through changing the duration and number of electrodeposition cycles.

**Figure 10:**
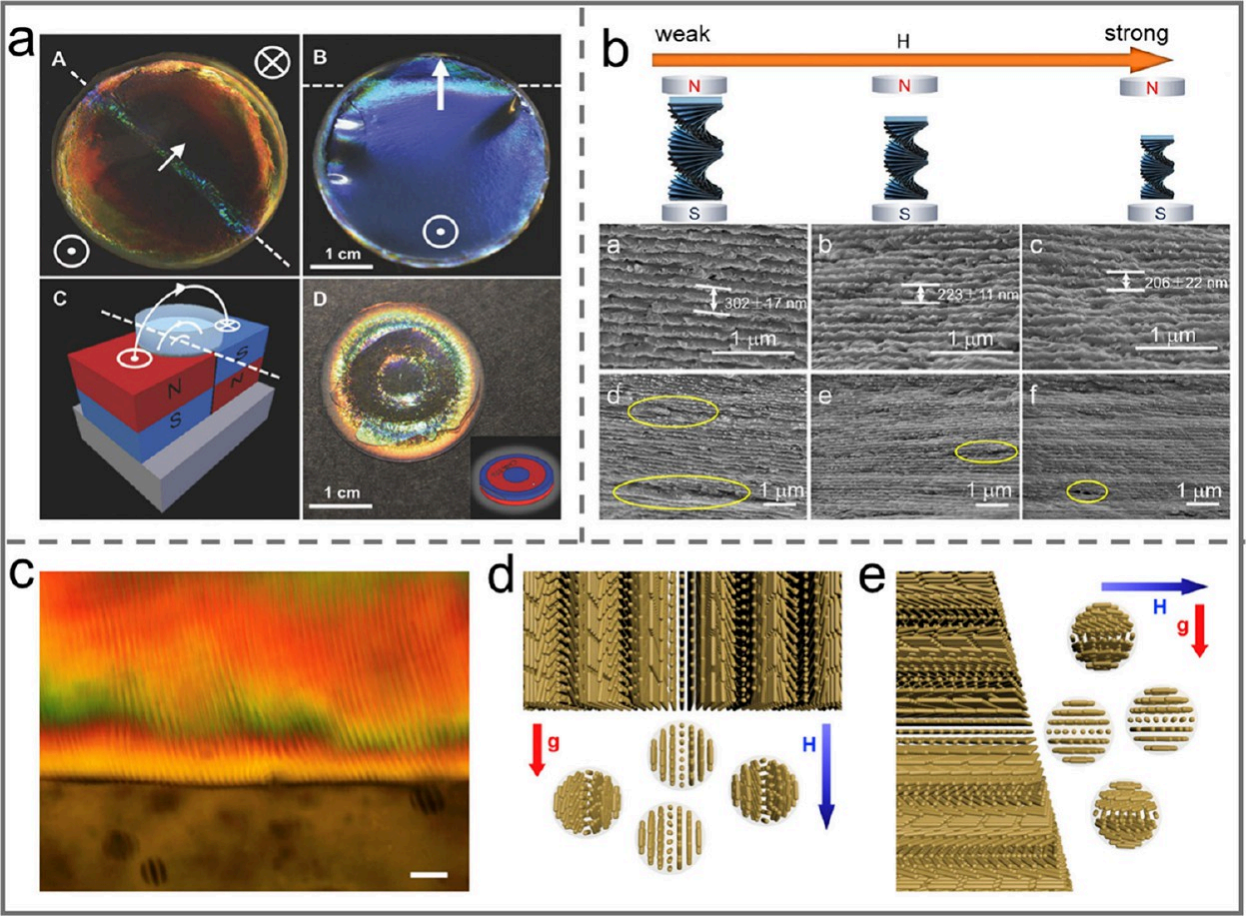
a) Films produced by slow evaporation of aqueous CNC suspension in a dish placed on NdFeB magnets. Reprinted with permission under a Creative Commons Attribution (CC BY) License from ref (140). Copyright 2017 Wiley-VCH. b) SEM images of CNC films under magnetic fields with varying strength. Reprinted with permission from ref (141). Copyright 2020 American Chemical Society. c) PLM image showing the phase separation of a CNC/MNP dispersion in a vertical gradient magnetic field. d) 3D model showing the phase separation and orientation of tactoids when the magnet was placed under the vial. e) 3D model showing the phase separation and orientation of tactoids when the magnet was placed on the right side of the vial. Reprinted with permission from ref (142). Copyright 2019 Elsevier.

Apart from an electric field, a magnetic field can also be employed to promote the alignment of the CNC nanorods. Note that the perpendicular alignment is locally induced due to the negative diamagnetic susceptibility of CNCs. Sugiyama et al. demonstrated that aqueous suspensions of CNC isolated from tunicate could be strongly oriented under a magnetic field of 7 T.^143^ The orientation of the crystal axis was perpendicular to the magnetic field direction due to the intrinsic anisotropic magnetic susceptibility of the individual C–C, C–O, C–H, and O–H bonds from CNC and their relative orientation in the crystal. Recently, De France et al. utilized SAXS to investigate how weak magnetic fields and CNC suspension concentration affect the kinetics and degree of CNC alignment.^144^ Results showed that partial alignment occurred with the CNCs suspension above the critical concentration under a 1.2 T magnetic field within 2 min. After that, a slower cooperative ordering was observed and finally resulted in the formation of an almost perfect alignment within 200 min. Such a near-perfect alignment also formed at a relatively low magnetic field of 0.56 T, but the ordering was 36% slower. However, for the suspensions below the critical concentration, no magnetic alignment was observed. Similarly, small commercial magnets (≈ 0.5–1.2 T) were used by Frka-Petesic et al. to tune the arrangement of the chiral nematic domains in suspension and to generate CNC films with structural colors (Figure 10a).^140^ When the surface of CNCs was decorated with magnetic nanoparticles, the strength of the magnetic field used to tune CNC self-assembly could be very low. For instance, Chen et al. reported that the self-assembly of the CNCs decorated with Fe_3_O_4_ nanoparticles could be facilely tuned using a small magnetic field of 7 mT.^141^ When the strength of the magnetic field increased from 7 mT to 15 mT, the pitch of the chiral nematic structure decreased from 302 to 206 nm (Figure 10b). However, an increase in pitch with increasing external magnetic force was observed in pure CNC. Zhang et al. prepared uniformly aligned flexible magnetic films with Fe_3_O_4_-nanoparticles-decorated CNCs.^145^ The original helicoidal organization underwent unwinding and transformed to uniaxial alignment under a magnetic field lower than 150 mT, resulting in the formation of an almost perfect orientation over a large area. The results demonstrated that the high shear rate of circular flow triggered by the magnetic field exceeded that of classical EISA by 2 orders of magnitude. Such a high shear rate promoted the nontraditional, unidirectional orientation and untwisting helical arrangement of nanorods along the gradient magnetic field. Moreover, the obtained films showed fast actuation and shape deformation under weak magnetic fields, local humidity changes, and photothermal-induced one-side stresses. Wang et al. explored the manipulation of lyotropic liquid crystal systems through the integration of superparamagnetic nanoparticles.^142^ By doping CNCs with these nanoparticles, a distinction was made between isotropic and liquid crystalline phases based on their differing magnetic susceptibilities. Utilizing gradient magnetic fields, the study demonstrated the spatial separation of these phases, with liquid crystalline tactoids migrating toward lower-field regions, while isotropic phases gravitate toward higher-field areas (Figure 10c–e). Furthermore, this technique enabled precise control over the positioning and orientation of chiral nematic liquid crystalline phases. In another study reported by MacLachlan and co-workers, tunable diffraction gratings were produced with vertically aligned uniform periodic structures by immobilizing highly oriented chiral nematic liquid crystals of CNCs in polymer networks using the synergistic effect of gravity on phase separation and magnetic field on liquid crystal orientation.^146^

**Figure 11:**
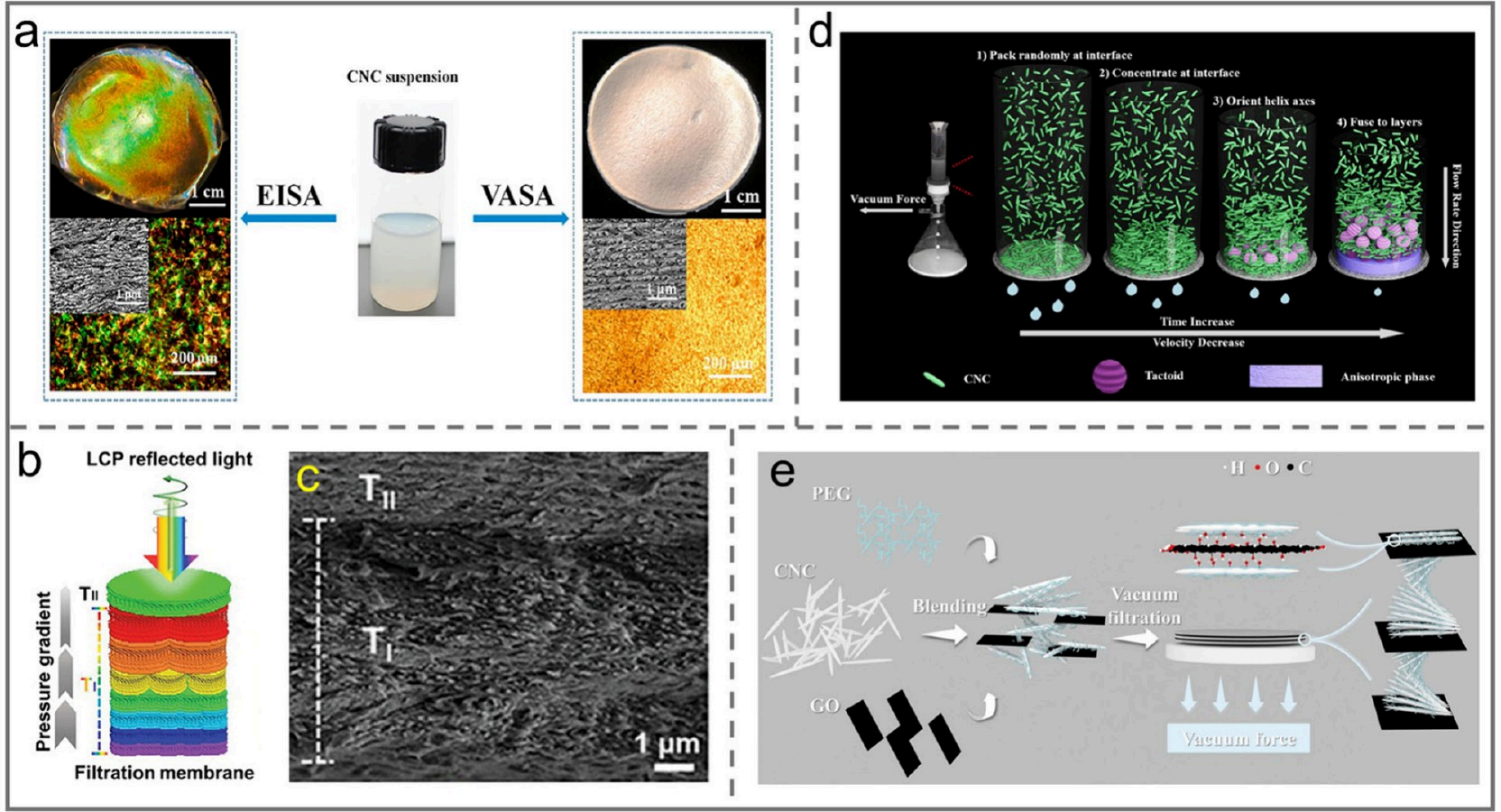
a) Photographs and PLM images of iridescent CNC films fabricated with the EISA technique (left) and the VASA technique (right). Reprinted with permission from ref (147). Copyright 2019, Elsevier. b) Schematic illustration of pressure-directed self-assembly of chiral pearlescent CNC films. c) Cross-sectional SEM image of the chiral pearlescent CNC films. Reprinted with permission from ref (94). Copyright 2023 Wiley-VCH. d) Schematic illustration of VASA of CNCs. Reprinted with permission from ref (148). Copyright 2020 Elsevier. e) Schematic illustration of the CNC/PEG/GO film fabrication process. Reprinted with permission from ref (149). Copyright 2024 Elsevier.

Although EISA has been widely used to fabricate iridescent films, there are still some limitations to this approach, such as a long fabrication time, color nonuniformity, high initial concentration, and uneven surface. Figure 11a shows the nonuniform color of the CNC film fabricated through EISA, indicating that the helical directions of CNC chiral nematic domains are randomly distributed.^147^ In contrast to EISA, vacuum-assisted self-assembly (VASA) can produce CNC films with more uniform color. Wang et al. reported the preparation of chiral pearlescent films with tunable optical properties from CNCs by using vertical pressure to direct their self-assembly (Figure 11b).^94^ Such vertical pressure was achieved by using a vacuum filtration cup, where CNCs and water were separated by a pressure difference created by a vacuum pump removing air from below the filtration membrane. The vertical pressure directed the CNCs to align perpendicular to the film plane, forming a densely stacked structure. Initially, the CNC suspension experienced a decreasing pressure, followed by a steady pressure on the remaining suspension, resulting in a bilayer film. The bottom layer had densely stacked microdomains with a vertical gradient pitch, while the top layer had a uniform pitch (Figure 11c). This vertical pressure filtration produced bilayer films with chiral pearlescence and a specular reflectivity. The mechanism of CNC self-assembly into chiral nematic structures during VASA was investigated by Zhang’s group.^148^ The structural evolution of CNC aggregations on filter paper was clarified by “freezing” the CNC suspensions in polyacrylamide matrices at different filtration stages. It was found that the self-assembly of CNC transferred from the isotropic phase to the chiral nematic phase at the paper/suspension interface (Figure 11d). Tactoids were formed in the concentrated CNC suspension near the filter paper. The VASA promoted the helical axes of the CNC tactoids along the flow-field direction, resulting in a faster formation of long-range ordered liquid crystals through nucleation growth. VASA, in comparison to EISA, is a less time-consuming approach. Thus, the precursor suspensions do not require long-term stabilization, which facilitates the coassembly of CNCs with other functional nanoparticles, such as graphene oxide (GO) and carbon nanotubes (CNTs). Li et al. also investigated the effects of two different film-making techniques, EISA and VASA, on the structural, optical and mechanical properties of the obtained iridescent CNC films.^150^ The results showed that the films obtained from VASA exhibited a smooth surface and a uniform and dense chiral nematic organization due to the strong pressure, while the films obtained from EISA exhibited a fish-scale-like surface and a more random chiral nematic structure with voids due to the multistage assembly. Consequently, VASA-CNC films exhibited a more uniform structural color and enhanced mechanical performance. CNC/PEG/GO composite films with superior UV-blocking and transparency were produced by Xia et al. through VASA (Figure 11e).^149^ The filtration process reduced the helical pitch of the CNCs’ chiral nematic structure, shifting the photonic bandgap to the UV region. This structure, combined with the UV absorption of GO, enabled efficient UV-blocking while maintaining transparency. Similarly, Ren et al. fabricated conductive chiral composite films by assembling a CNC host matrix with multiwalled carbon nanotubes (MWCNTs).^151^ The addition of MWCNTs had an important impact on the chiral nematic structure of the prepared nanopaper. With the increase in the content of MWCNTs, the chiral nematic structure of the composite shifted from left-handed to right-handed.

**Figure 12:**
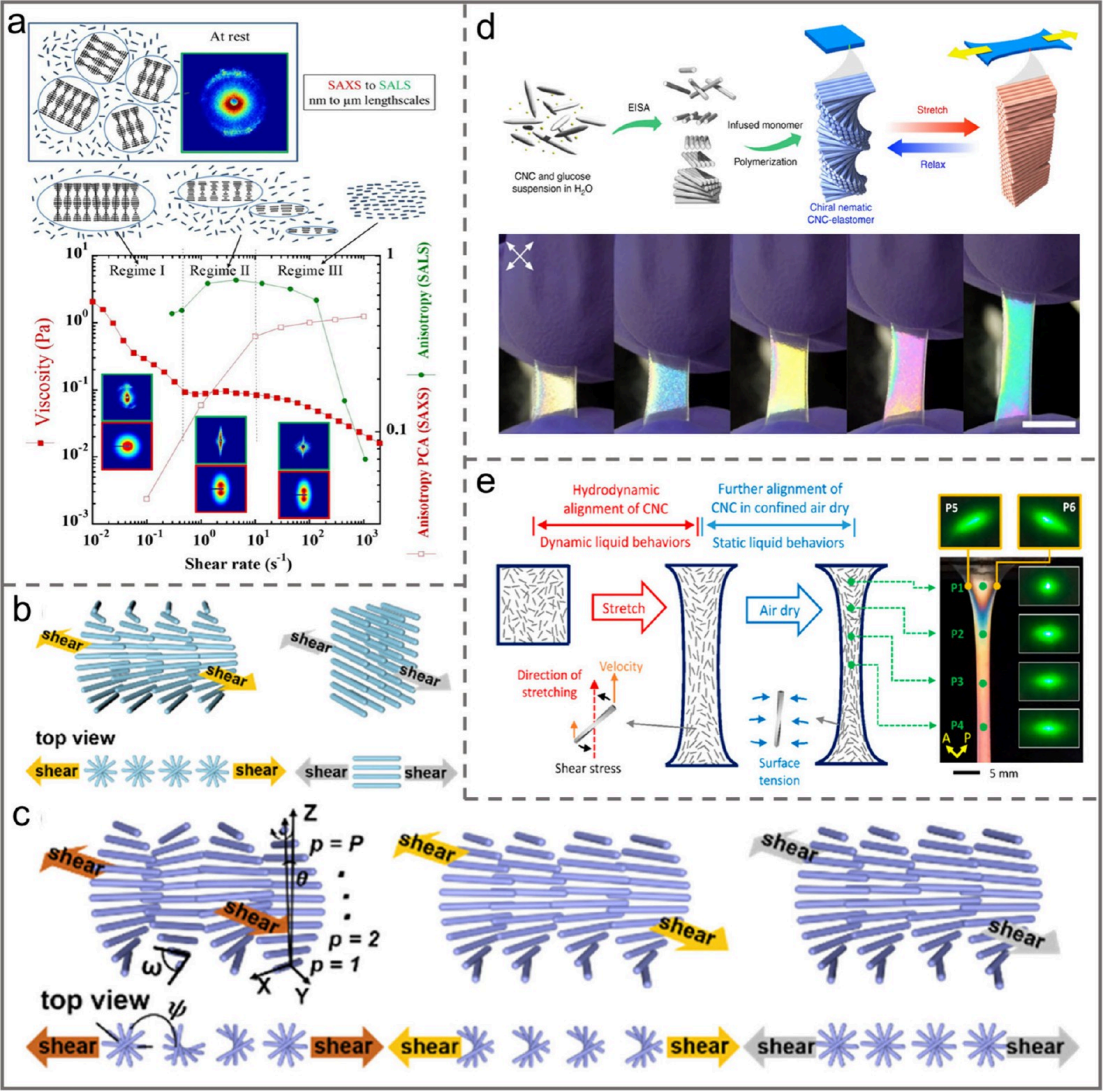
a) Schematic illustration of CNC suspensions from rest to increasing applied shear rates. Reprinted with permission from ref (152). Copyright 2021 Elsevier. Schematic illustration of the reorientation of CNCs by shear flow b) in-plane unwinding process and c) later vertical helical unwinding process. Reprinted with permission from ref (153). Copyright 2023 American Chemical Society. d) The CNC particles were reoriented from a chiral nematic state to an aligned structure via stretching. Reprinted with permission under a Creative Commons Attribution (CC BY) License from ref (7). Copyright 2019 Springer Nature. e) Schematic illustration of the hydrodynamic alignment of CNCs in dynamic hydrogels under stretching. Reprinted with permission from ref (154). Copyright 2019 American Chemical Society.

Shear stress applied to the CNC suspensions during the drying process also greatly affects the self-assembly behavior of these nanocrystals and the texture of the CNC films. Pignon and co-workers investigated the underlying mechanisms of structural evolution in concentrated CNC suspensions under shear and during relaxation postshear using small-angle X-ray scattering, light scattering, and rheometry.^152^ They linked dynamic structural changes across nanometer to micrometer scales to the well-known three-regime rheological behavior. The first shear-thinning part, regime I at lowest shear rate, is ascribed to the progressive disruption of liquid crystalline domains into micrometer-sized tactoids aligned toward the velocity direction with an internal cholesteric organization of the CNCs and the helical axes oriented vertically (Figure 12a). In the intermediate shear rate domain, regime II, upon reaching the viscosity plateau, the micrometer-sized tactoids break down into elongated tactoids with a critical size even smaller than the pitch value, oriented along the velocity direction. Regime III at high shear rate was associated with the parallel flow of individual CNCs along the velocity direction. Feng et al. investigated the tailoring of the chiral nematic organization of CNCs via shear flow through complete polarization analysis using Mueller matrix microscopy.^153^ An early in-plane unwinding followed by a later helically vertical unwinding was proposed as the main process of the self-assembled structure changes under shear flow (Figure 12b and c). Sufficient shearing is necessary for the reorientation of the helically aligned CNCs to form the nematic structures. All in all, the structure of CNC films prepared from chiral nematic CNC suspensions is sensitive to the shear stress applied during the drying process. Applying a suitable shear stress is thus crucial to produce a photonic film with desirable optical properties.

Mechanical stress induced by both stretching and compressing was also employed for tailoring the pitch and the alignment of the resulting nanostructure. Applying tensile load facilitated the alignment of CNC along the direction of the induced stretch. Such alignment was detected in both wet and dry all-cellulose nanocomposites under high strain conditions.^7,154^ However, note that it is not feasible to change the alignment of CNCs by mechanical stretching within pure CNC films due to their brittle nature. Incorporating CNCs into elastomers is an effective way to produce highly stretchable composites with aligned CNCs. For instance, Kose et al. prepared a highly stretchable and homogeneous CNC/elastomer composite film with a chiral nematic structure. This structure could be easily tuned by stretching and relaxing (Figure 12d).^7^ The chiral nematic structures transformed into pseudonematic structures when the composite film was stretched. The composite film displayed bright interference colors between crossed polarizers in response to stretching and relaxing. It was also found that when stretching was applied parallel to the direction of CNC alignment, the arrangement of CNCs was further enhanced and the birefringence of the film was increased.^155^ On the contrary, when stretching was applied perpendicular to the direction of CNC alignment, the arrangement of CNCs became more disordered and the birefringence of the film was decreased. Such a composite can be used to fabricate reversible stimuli-responsive materials for applications in flexible optics and sensors. Dynamic hybrid hydrogels with highly variable birefringence were prepared by utilizing the hydrodynamic alignment of CNCs through the shear-thinning phenomenon in the hydrogel network (Figure 12e).^154^ The hydrodynamic aligned structure could be preserved when the stretching was terminated due to the fact that the reconstruction of hydrogel networks was much faster than the dissipation of CNCs orientation. Moreover, the surface tension of hydrogels during the drying process further enhanced the orientation index of CNC, resulting in anisotropic behavior with a tunable birefringence.

**Figure 13:**
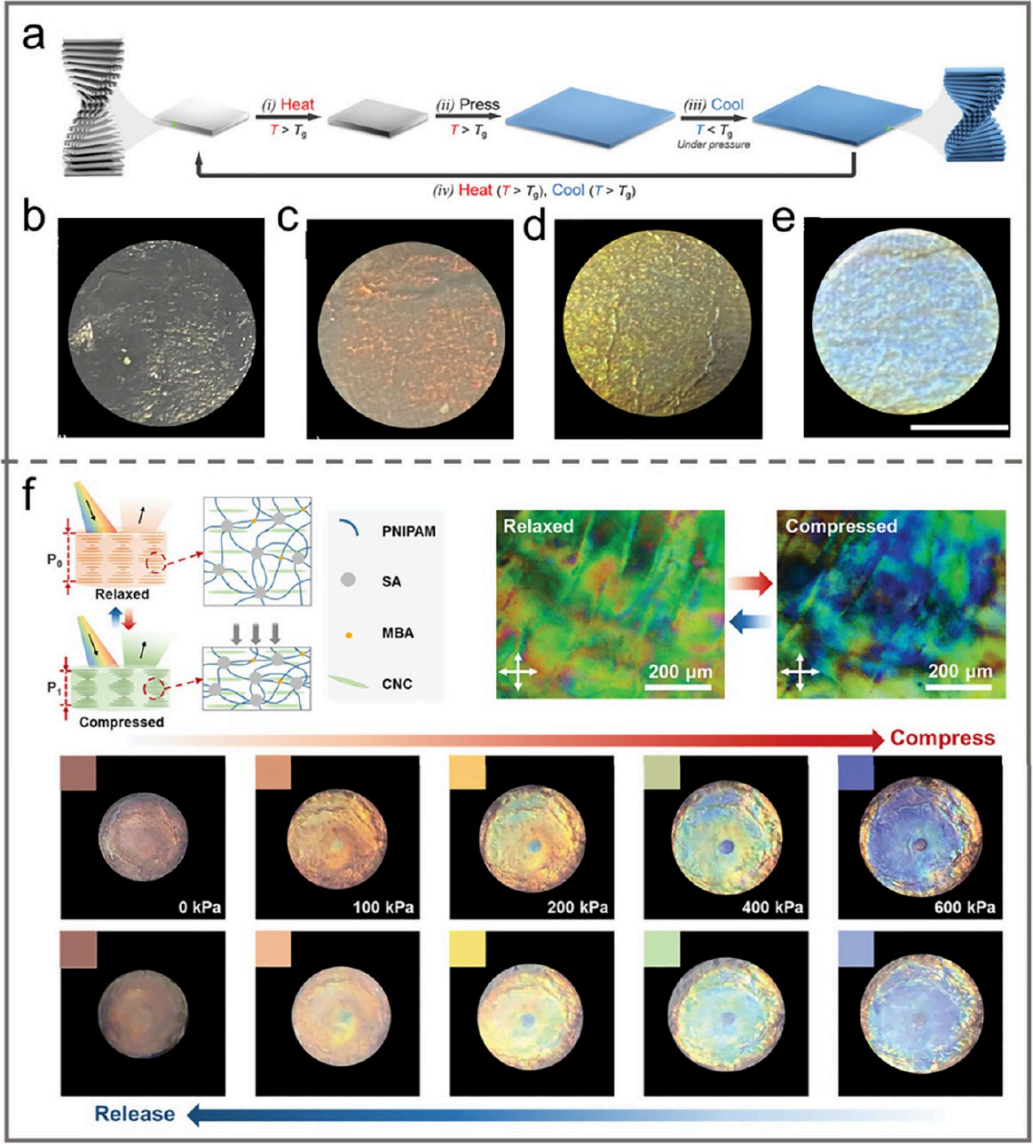
a) Schematic illustration of the sequential programming and recovery of CNC-SMP. Photos of the CNC-SMP pressed at b) 0 N, c) 140 N, d) 180 N, and e) 230 N. Reprinted with permission from ref (156). Copyright 2021 Wiley-VCH. f) Pressure-responsive property of the hydrogel. Reprinted with permission from ref (157). Copyright 2023 Wiley-VCH.

Similarly, the alignment of CNCs can also be tuned by applying mechanical compression. For instance, MacLachlan et al. reported the fabrication of a shape-memory photonic crystal thermoplastic which allows reversible capture of different colored states by embedding chiral nematic CNCs into a polyacrylate matrix.^156^ After equilibration at a temperature above its glass transition temperature, e.g. 100 °C, pressure was applied to the resultant thermoplastic composite. Then it was cooled down to room temperature while maintaining the applied pressure (Figure 13a). Upon removal of the pressure, the sample could retain its deformed shape. By simply increasing the pressure applied, the structural color could be tailored from red to blue due to compression of the helical pitch of the CNC chiral nematic structure. Moreover, heating the composite to 100 °C caused it to return to its original shape (Figure 13b–e). By taking a similar strategy, the CNC self-assembled chiral nematic structure was embedded into a kind of thermosensitive copolymer, i.e, poly(isopropylacrylamide-*co*-stearyl acrylate) to prepare a pressure/temperature dual-responsive hydrogel.^157^ When the resultant hydrogel was subjected to a gradually increasing external pressure, the chiral nematic structure embedded in the composites was correspondingly compressed in the vertical direction so that the helical pitch decreased, which resulted in a blue shift of the structural color (Figure 13f). Moreover, owing to the thermoresponsive molecular structures of the polymer matrix, as the sample was heated from 25 °C, 35 °C, 45 to 55 °C, the main structural color correspondingly changed from red, orange, yellow to blue because of the decrease of the helical pitch.

Except for the above external factors, the supported substrate was also verified as an important parameter that can be employed to tune the pitch of the CNC-based chiral films. Nguyen et al. investigated the influence of different supported substrates, including aluminum, alloy steel sheet, acid-treated silicon wafer, mica, cellulose acetate, polystyrene, Teflon, paraffin, polyethylene, nitrocellulose, and a polydimethylsiloxane (PDMS)-coated glass plate, on the formation of CNC films.^158^ A photonic pattern in the CNC film showing almost colorless letters of “NCC” on a blue background was successfully achieved by changing the substrate to adjust the pitch. The almost colorless region had a chiral nematic structure and reflected NIR light. It is worth noting that the substrate affects the thickness of the films due to the apparent change in the helical pitch.

The introduction of a nonvolatile solute also greatly affects the equilibrium pitch of the chiral nematic structure. Mu et al. found that the addition of d-(+)-glucose resulted in a decrease in the pitch of chiral nematic suspensions, but an increase in the pitch of the dry films and therefore a red-shift reflection band.^90^ Results showed that there were two different stages of changing the pitch during the evaporation process. In the first stage, the pitch decreased with the increase of concentration due to evaporation. At this stage, the addition of glucose caused a decrease in the pitch. In the second stage, with the evaporation of the solvent, the concentration of CNCs reached where the formation of ordered gels and glasses occurred, thus preventing further major changes in pitch. At this stage, the addition of glucose lowered the CNCs concentration and led to an increase in pitch.

## 4 INFLUENCE AND REGULATION OF LIQUID CRYSTAL BEHAVIORS OF ChNCs

ChNCs, like CNCs, can self-assemble into a chiral nematic phase with the critical concentration being a key factor in this process. Initially, when the CNC volume fraction exceeds a critical value (ϕ_0_), the chiral nematic phase coexists with the isotropic phase. As the concentration increases further, the chiral nematic phase grows until the suspension is entirely chiral nematic at a second critical volume fraction (ϕ_1_). Importantly, these critical concentrations (ϕ_0_ and ϕ_1_) are inversely proportional to the particle aspect ratio. Fungi-derived ChNCs have a higher aspect ratio than shrimp-derived ChNCs when prepared under similar hydrolysis conditions.^92^ As expected, they exhibited lower biphasic threshold concentrations and a narrower biphasic region. Additionally, fungi-derived ChNC suspensions had significantly lower pitch values compared to shrimp chitin suspensions at similar concentrations.

**Figure 14:**
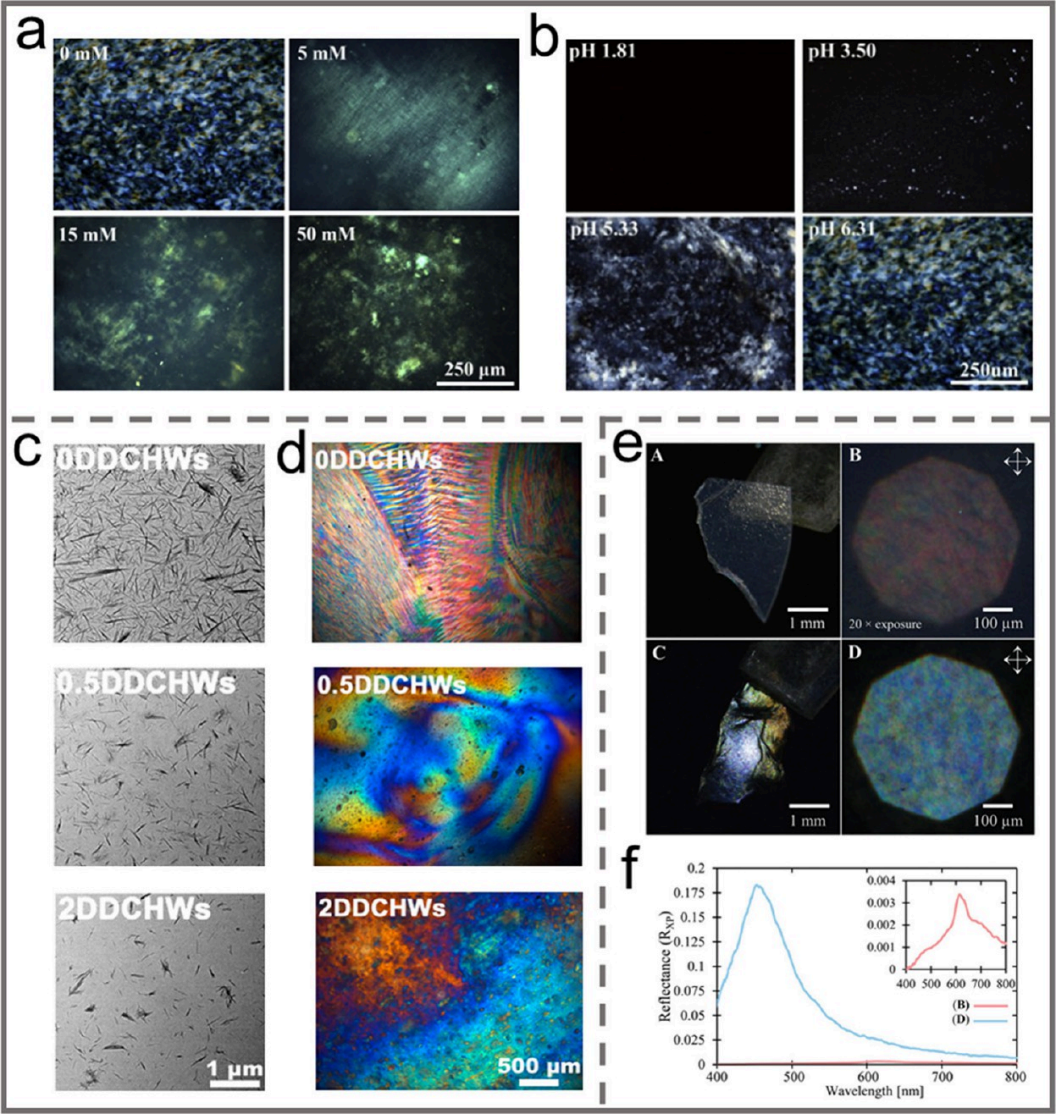
a) PLM images of 5.0 wt % ChNC aqueous suspension with different salt concentrations. b) PLM images of 5.0 wt % ChNC aqueous suspensions with different pH values. Reprinted with permission from ref (159). Copyright 2019 Elsevier. c) TEM images of ChNCs with different deacetylation time. d) PLM images of ChNC suspensions. Reprinted with permission from ref (160). Copyright 2023 American Chemical Society. e) Photograph and optical microscopy image of the ChNC film before (A, B) and after alkaline treatment (C, D). f) Reflection spectra under crossed polarizers of (B) and (D). Reprinted with permission under a Creative Commons Attribution (CC BY) License from ref (92). Copyright 2022 Wiley-VCH.

Previous experiments have confirmed that the self-assembly of ChNCs is greatly influenced by many other internal and external factors.^22,54^ Depending on the isolation approaches, diverse functional groups can be introduced onto the surface of ChNCs, resulting in different surface charges. Tuning electrostatic interaction by changing the pH value or ionic strength of ChNC suspensions has been extensively studied and employed as an effective way to tailor their self-assembly process.^160^ For instance, Luo et al. reported an electrolyte-free ChNC suspension with a concentration of 5 wt % without exhibiting a highly ordered chiral nematic structure (Figure 14a).^159^ The ordered structure can be partially retained at a concentration of 5 mM NaCl. With a further increase in the NaCl concentration, the suspension transformed from a liquid crystal phase to a colloid glass state and then to an elastic gel state. Increasing the pH value of the suspension leads to deprotonation of the surface amine groups of ChNCs, thus resulting in changes in the interactions between these nanocrystals. Results showed that no birefringence could be observed in the 5 wt % ChNCs suspension at a pH of 1.81 (Figure 14b). When the pH was 3.5, randomly distributed decentralized liquid crystal domains were observed (Figure 14b). As the pH increased further, the birefringent structure of the suspensions became increasingly apparent. This could be explained by the fact that at a low pH value, e.g., pH 1.8, all the amine groups were protonated, resulting in strong electrostatic repulsion between these nanocrystals. As the pH increased, the amino groups were deprotonated, reducing the nanocrystal–nanocrystal electrostatic repulsion. Therefore, the nanocrystals were arranged in an orderly manner with enhanced orientation. Note that ChNCs synthesized with different reactions generally have distinct surface physicochemical properties, and thus, they may exhibit different self-assembly behaviors at the same pH condition. The same group reported in another study that ChNC suspensions with pH ranging from 3 to 11 exhibited fingerprint-like liquid crystal texture.^160^

Deacetylation of ChNCs, generally achieved via NaOH treatment, causes the elimination of acetyl groups and the exposure of amine groups, thus influencing their self-assembly behavior and liquid crystalline features. ChNCs can maintain the crystalline structure well after NaOH treatment for a certain period. However, with the increase of the deacetylation time, the aspect ratio of the nanocrystals decreased (Figure 14c), the zeta potential increased, and the fingerprint-like texture progressively vanished.^160^ Compared with the pristine ChNC suspension, only sparse liquid crystalline domains were observed for the ChNC suspensions deacetylated for 2 h, indicating the disruption of the well-ordered Bougliand structure (Figure 14d). In-situ deacetylation was employed to increase the birefringence of the ChNCs photonic film, which was fabricated by slow-evaporation of suspensions of ChNCs isolated from mushrooms.^92^ Although the pitch of the photonic films was in the range to reflect visible color, unlike CNC photonic films prepared by using the same methods, the resultant films appeared macroscopically transparent, and only weak coloration was observed using polarized microscopy (Figure 14e). To enhance the birefringence of the photonic films, an in situ deacetylation treatment was applied to the photonic film. The deacetylated photonic film showed an obvious blueshift in the reflected color and a surprising 50-fold enhancement in the reflected intensity (Figure 14f), which was ascribed to the decrease in the pith due to the elimination and solubilization of the acetyl groups.

Tip sonication has been widely employed as a posthydrolysis treatment to disaggregate large bundles of ChNCs, leading to well-dispersed ChNC suspensions. Silvia investigated the effect of sonication energy input on the final properties of the ChNC suspensions and their liquid crystal phase behavior.^22^ As expected, tip sonication reduced the dimensions and polydispersity of ChNCs, but it did not significantly affect their surface charges. The ChNC suspensions treated with various sonication energy inputs exhibited comparable threshold concentrations at which they separated into two liquid crystalline phases and then into a fully chiral nematic phase. However, the chiral nematic pitches of the samples varied considerably. The ChNC suspension treated with higher energy input possessed a larger pitch at low nanocrystal concentrations and showed a steeper decrease in the pitch with increasing ChNC concentration. This could be explained by the fact that the ChNC bundles, characterized by a strong chiral shape and twisting power in the anisotropic suspensions, were broken into smaller rod-like units, leading to a smaller induced twist between ChNCs and, consequently, a larger pitch.

Li et al. investigated the effect of the surface charge of ChNCs on the formation of the chiral nematic phase.^161^ By N-sulfonation of the amino groups, the surface charge of ChNCs was changed from positive to negative without affecting the axial ratio of the crystallites. When the N-sulfonation reached S/N > 3, the N-sulfonated ChNCs formed a chiral nematic phase. As the N-sulfonation increased to S/N = 5, tactoids with a chiral nematic pitch of about 40 μm were found (5 wt %, pH 7). The surface charge of ChNCs can also be changed from positive to negative after hydrolyzation with H_2_O_2_, allowing the resultant nanocrystals to disperse well in alkaline or neutral aqueous media.^162^ It was found that with the increase in the ChNC concentration, the dispersion showed a typical lyotropic phase transition from anisotropic monophase to birefringent mesogence and then gelation. Furthermore, the sol–gel transition of ChNCs was influenced by the surface charge density, concentration, temperature, and aging time.

**Figure 15:**
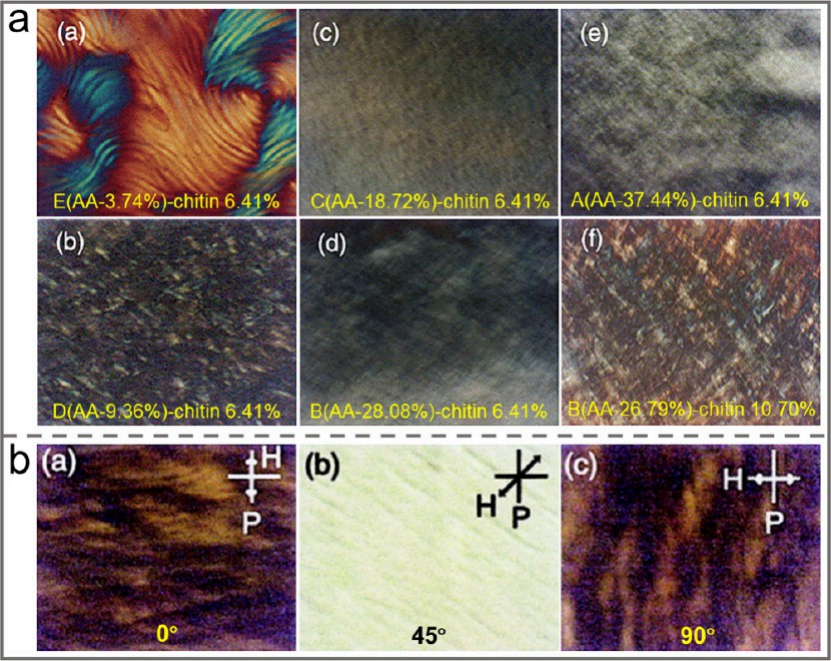
a) PLM images of ChNC/AA composite with different concentration of ChNC and AA. Reprinted with permission from ref (163). Copyright 2003 American Chemical Society. b) PLM images of a ChNC/PAA (55:45) composite. Reprinted with permission from ref (164). Copyright 2003 Wiley-VCH.

Similar to what has been reported for CNC-based self-assembly materials, adding additives and applying external energy fields are efficient strategies for regulating the optical properties of ChNC films. Nge et al. studied the liquid crystal phase behavior of ChNC/acrylic acid (AA) liquid mixture.^163^ The concentration required to form a complete chiral nematic phase in the ChNC/AA mixture was higher than that in pure ChNCs in water. The high concentration of AA increased the viscosity, thus hindering the phase separation of the mesophase. The fingerprint-like patterns, characteristic of chiral nematic textures, were observed only at low AA concentrations (Figure 15a). After the addition of AA, a stable flow-birefringence glassy phase was observed within a narrow ChNC concentration range of approximately 6.22–6.41%. This phase yielded a clear boundary between the isotropic phase and anisotropic phase. The composite film of ChNC/poly(acrylic acid) (PAA), which was magnetically aligned and optically anisotropic, was achieved by applying a high magnetic field of 5 T.^164^ It was found that the orientation of the optical texture preferred to be perpendicular to the direction of the applied magnetic field (Figure 15b). From the results of PLM and XRD, the composite with a higher ChNC concentration (10.70 wt %) exhibited a high degree of crystallite orientation of 0.70. However, at a certain concentration of AA, the degree of orientation decreased with decreasing ChNC concentration.

## 5 APPLICATIONS OF SELF-ASSEMBLED PNs FUNCTIONAL MATERIALS

The self-assembly behavior of PNs is attractive for various potential advanced applications. In this section, we mainly introduce recent advances in self-assembled PN functional materials and their promising applications.

### 5.1 Stimuli-Responsive Photonic Materials

Recently, stimuli-responsive photonic materials have received widespread attention owing to their giant potential in information storage materials, sensors, intelligent devices, etc. A variety of spectral features of CNCs- and ChNCs-based structural color materials offer great opportunities for designing and fabricating stimuli-responsive photonic materials via diverse approaches. For instance, rich surface hydroxyl groups on CNCs and ChNCs allow easy functionalization with various functional groups that can respond to external stimuli such as humidity, pH, temperature, or specific gases.^32^ Additionally, stimuli-responsive photonic materials could be fabricated by directly adding stimuli-responsive components into the chiral nematic structures formed by CNCs or ChNCs.^31^

**Figure 16:**
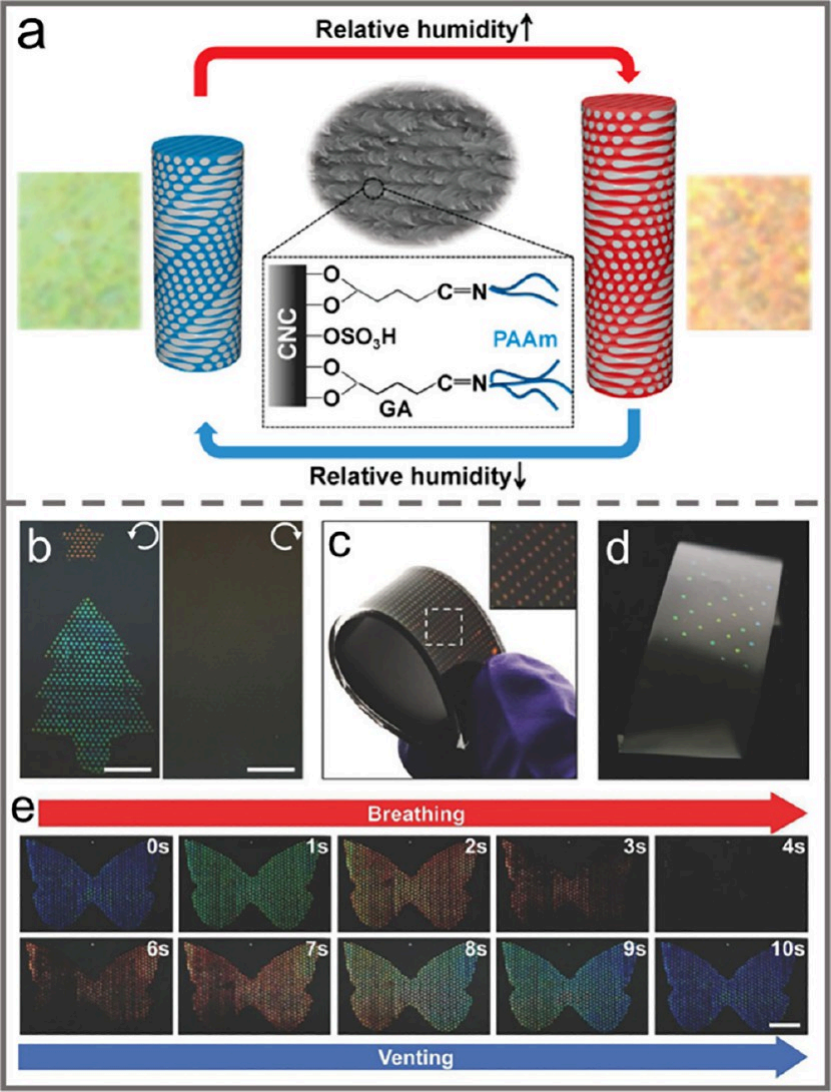
a) Schematic diagram showing the structure of iridescent films changed with humidity. Reprinted with permission from ref (165). Copyright 2017, American Chemical Society. b) Dot matrix image of a “Christmas tree” observed by circular polarizer. c) CNC microfilm obtained on a flexible PDMS substrate. d) CNC photonic array transferred to adhesive tape. e) Response of CNC microfilms to humidity changes. Reprinted with permission under a Creative Commons Attribution (CC BY) License from ref (166). Copyright 2019, Wiley-VCH.

It has been found that the iridescent color of self-assembled CNC films can be adjusted by controlling the humidity. The sorption–desorption of water leads to changes in the chiral nematic pitch, thereby altering the reflectivity of the CNC film. Lu et al. fabricated a high-performance photonic humidity-responsive sensor consisting of CNCs, polyacrylamide, and glutaraldehyde (Figure 16a).^165^ When the relative humidity increased from 11% to 97%, the pitch of the chiral nematic structure enlarged due to the water-induced swelling of the polyacrylamide matrix. As a result, the color of the composite changed from green to red. In a similar way, Yao et al. reported the fabrication of flexible CNC/PEG composite films with homogeneous structural color. The regulation of the structural color could be realized by adjusting the composition of CNCs and PEG or by changing the relative humidity of the external environment.^6^ Due to the reversible swelling and dehydration of the chiral nematic structure, the composite film of CNC/PEG (80/20) displayed a reversible structural color change from green to transparent at varying relative humidity from 50% to 100%. Additionally, the composite also exhibited superior thermal and mechanical properties, endowing it with attractive versatility. Zhao et al. developed a scalable printing method to produce arrays of structurally colored CNC microfilms from spatially defined nanoliter sessile droplets (Figure 16b and c).^166^ Arrays can be transferred to a wide range of substrates using adhesive tape to separate them (Figure 16d). The resulting films exhibited remarkably homogeneous and intense color, resulting in an extremely consistent optical appearance across the array and a significant response to variations in humidity (Figure 16e). The printed CNC microfilm arrays have wide application prospects in interactive pigments, cosmetics, and anticounterfeiting applications.

**Figure 17:**
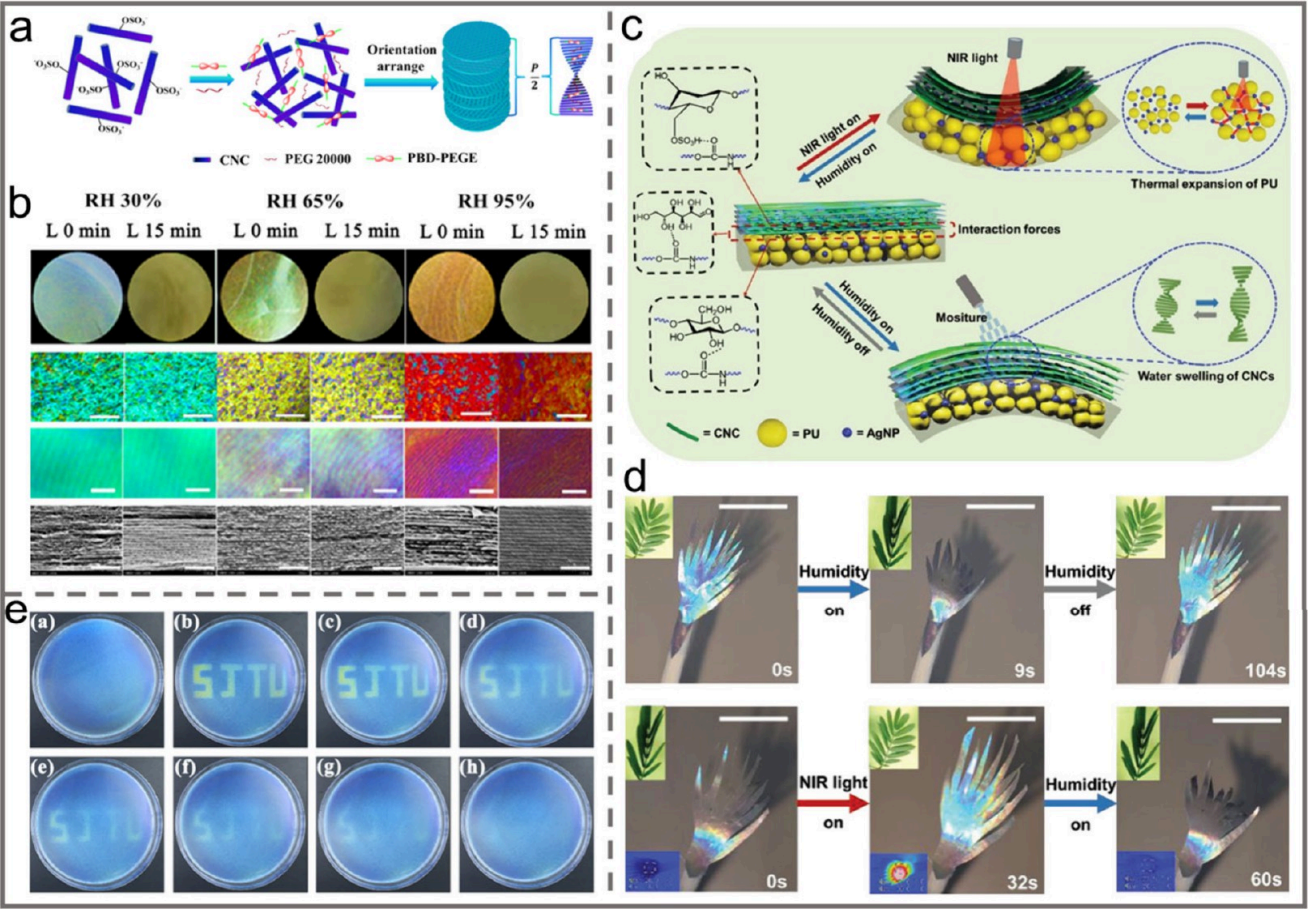
a) Schematic diagram of the coassembly process of the CNC-based composite films. b) Response of composite films to light at different relative humidities. Reprinted with permission from ref (167). Copyright 2020 American Chemical Society. c) Schematic diagram of the production of bioinspired composite films. d) A smart “mimosa” responded to moisture and NIR light. Reprinted with permission under a Creative Commons Attribution (CC BY) License from ref (168). Copyright 2021 Wiley-VCH. e) CNC composite film was used as humidity-responsive color-changing paper. Reprinted with permission from ref (169). Copyright 2019 American Chemical Society.

Multistimuli-responsive CNC-based chiral materials have been fabricated to broaden the scope of applications of CNC-based chiral nematic materials. For instance, light- and humidity-responsive chiral nematic photonic crystal films were fabricated by the coassembly of CNCs and poly(3,3′-benzophenone-4,4′-dicarboxylic acid dicarboxylate polyethylene glycol) ester (PBD–PEGE) with the addition of PEG (Figure 17a).^167^ After light irradiation under different relative humidities (RHs), the composite films preserved the chiral nematic and helical lamellar structures (Figure 17b). This dual-stimuli response of the composite films was related to the synergistic effects of the photoactive benzophenone groups of PBD–PEGE and the water-induced swelling of the hydrophilic polymer. A bioinspired bilayer actuator with reversible color- and shape-changing capabilities was fabricated by taking advantage of the CNCs-based self-assembled chiral nematic structures and their water or temperature response behaviors (Figure 17c).^168^ In the bilayer film, the soft polyurethane elastomer layer served as a self-assembly platform for CNCs to increase the flexibility of the material. Additionally, to achieve the ideal photothermal effect of the elastomer layer, AgNPs were added due to their high photothermal conversion efficiency. The actuator obtained with a synergistic iridescent appearance exhibited superfast actuation responses to humidity and near-infrared light (Figure 17d). Sun et al. successfully constructed humidity and heat dual response composite films by simply mixing hydrazone-groups-modified poly(*N*-isopropylacrylamide) (PNIPAM) copolymers and CNCs.^169^ The structural color of the films could be easily tuned by humidity, heat, or the content of the modified PNIPAM copolymers. More importantly, the change in color can be easily discriminated by the naked eye. Under high humidity conditions, water adsorption caused the resin to expand in volume, leading to a redshift. However, at a temperature higher than the lowest critical solution temperature of PNIPAM, the resin shrank, resulting in a blueshift. As shown in Figure 17e, the films displayed clear words under steam stimulation. Furthermore, the films demonstrated outstanding stability and cyclability to steam or liquid water owing to the strong chemical bonding between the resins and CNCs. Fan et al. fabricated visible multiresponse electrochemical sensors by simply mixing polyaniline (PANI) and CNC suspensions.^170^ The introduction of PANI not only offered a dark background to make the structural color of CNC more visible but also improved the conductivity of the composite film. The resulting films displayed potential applications in the field of visible electrochemical multisensing due to their responsiveness to pH, humidity, and organic solvents as well as changes in reflection wavelength, visible structural color, and conductivity. Meng et al. developed photonic films with multistimuli response behaviors to humidity, pH, and ethanol concentration by the coassembly of CNCs, sorbitol, and anthocyanin.^171^ The obtained photonic films showed significant color changes in response to a relative humidity change in the range of 50% to 100% or a pH change in the range of 2 to 12. Additionally, under different ethanol concentrations, these films displayed a fast response and good reversibility.

**Figure 18:**
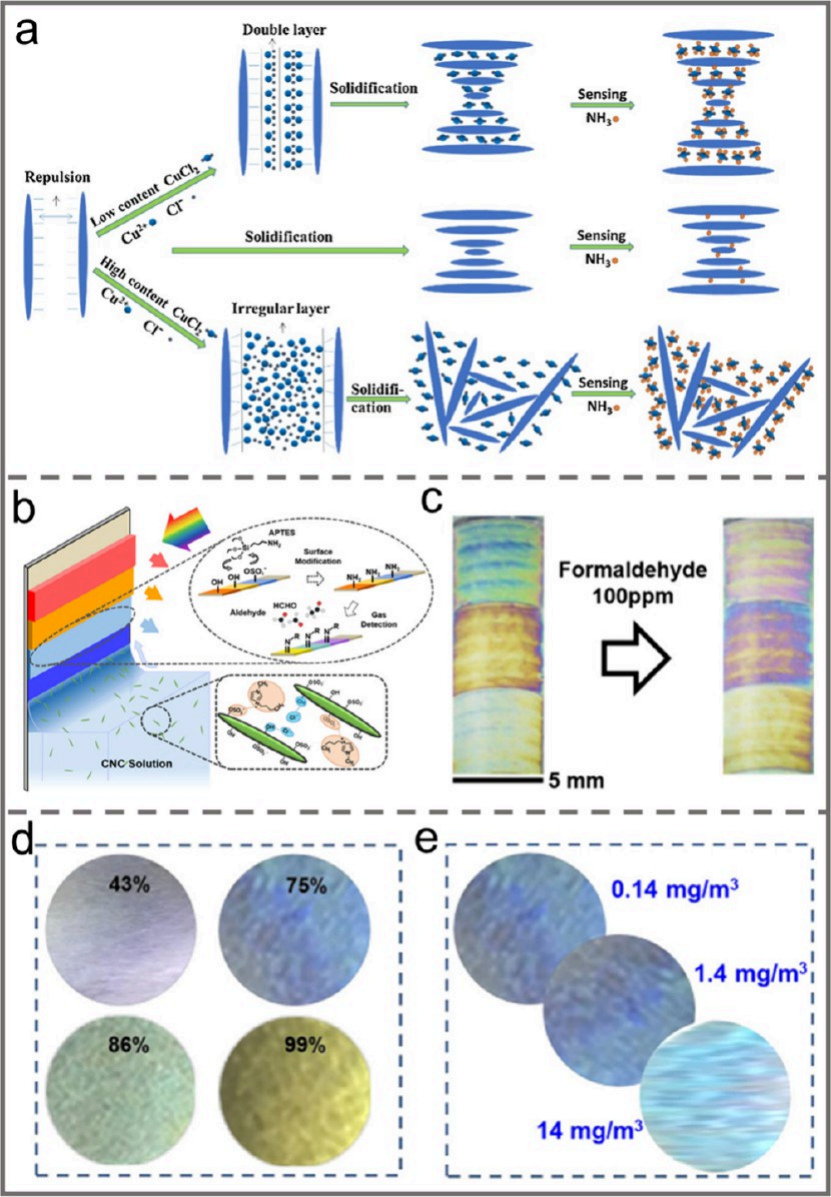
a) Schematic diagram of the structure and color-tuning mechanism of CNC or CNC-Cu(II) films. Reprinted with permission from ref (172). Copyright 2017 Elsevier. b) Schematic diagram of the production of the CNC-based films. c) Images of a CNC colorimetric sensor before (left) and after (right) exposure to formaldehyde at a concentration of 100 ppm. Reprinted with permission from ref (173). Copyright 2018 American Chemical Society. d) Optical images of a CNC film at different RHs. e) Optical images of a CNC film at different concentrations of formaldehyde gas. Reprinted with permission from ref (174). Copyright 2020 American Chemical Society.

Dai et al. designed a simple, inexpensive, and highly sensitive colorimetric sensor for detecting ammonia gas by doping copper salt into chiral nematic CNC film (Figure 18a).^172^ Since copper ions have a strong chelating affinity to ammonia gas, the composite film with a copper ion loading of 125 mmol/g showed remarkable sensitivity to ammonia gas. The composite film displayed a maximum redshift of 75 nm when the copper ion loading increased to 225 mmol g^–1^. Of note, excessive copper ions also have several negative effects on the chiral nematic CNC film, including salt microparticle deposition, which destroys the internal chiral nematic structure. Subsequently, Song et al. developed a CNC-based colorimetric sensor that changes color upon exposure to aldehyde gases, for example, propanal and formaldehyde.^173^ A layer of close-packed CNC was first deposited onto a silicon substrate by a dip-and-pull process with the aid of (1-butyl-3-methylimidazolium) molecules that can shield the electrostatic repulsion between CNCs. The thickness of the CNC layer could be easily adjusted by changing the pulling speed, thus giving rise to films with various colors in the visible range. The obtained CNC films were then chemically functionalized with amine groups. The films underwent obvious physical and chemical changes such as swelling as they were exposed to aldehyde gases. The color change could be readily observed with the naked eye at high concentrations of aldehyde gas or detected by a digital camera at parts per million levels (Figure 18b and c). Zhao et al. fabricated a dual-stimuli-responsive chiral nematic iridescent CNC film that exhibited a reversible response to environmental humidity and formaldehyde gas (Figure 18d and e).^174^ The dual response of the iridescent CNC films was related to the synergistic effect of cooperation and competition between water and the formaldehyde molecules. The humidity sensitivity of iridescent CNC films could be modulated by exposing the films to formaldehyde gas. Similarly, the formaldehyde sensitivity of the iridescent CNC film was also greatly affected by the humidity.

Although there are a large number of reports on the use of CNC to construct stimuli-responsive materials, little work has been done on combining stimuli-responsive materials with chiral nematic ChNCs. In 2022, Lee et al. designed a kind of anisotropic stimuli-responsive microgels depending on the chiral nematic phase of ChNCs and NIPAM.^27^ The microgels with controlled dimensions were prepared by emulsifying the mixture of ChNC suspension, NIPAM, the cross-linking agent, and initiator in a microfluidic device. After polymerization, the chiral nematic structures formed by ChNCs were embedded in the hydrogel matrix. The resultant microgel showed an asymmetric response to temperature, resulting in an obvious change in shape and optical properties. Additionally, the obtained chiral nematic structures possess the ability to deform in a constricted space. This study provides design guidance for biopolymer composites with programmable motion.

### 5.2 Photonic Encryption

As mentioned above, structural colors generated by the periodic variance of the refractive index have many merits such as nonfade character, high color saturation, and angle-dependent reflection. By taking advantage of the structural color features, complicated information can be encoded into the chiral nematic CNC- or ChNC-based functional materials for anticounterfeiting and/or encryption applications. For instance, inspired by the skin of chameleons, Wang et al., fabricated highly flexible and free-standing PEG/CNC composite structural color films.^175^ A reversible structural color change of the resulting films upon stretching could be observed by the naked eye. Moreover, the composite films showed compressive and water-responsive properties, allowing them to be used as photonic paper. At an RH of 56%, the characters written on the film by ink-free writing were yellow and showed clear outlines. As the RH increased to 100%, the whole film exhibited a slightly reddish translucence appearance and the characters were visible. The marks could be viewed again when the RH decreased to 56% and 16%.

**Figure 19:**
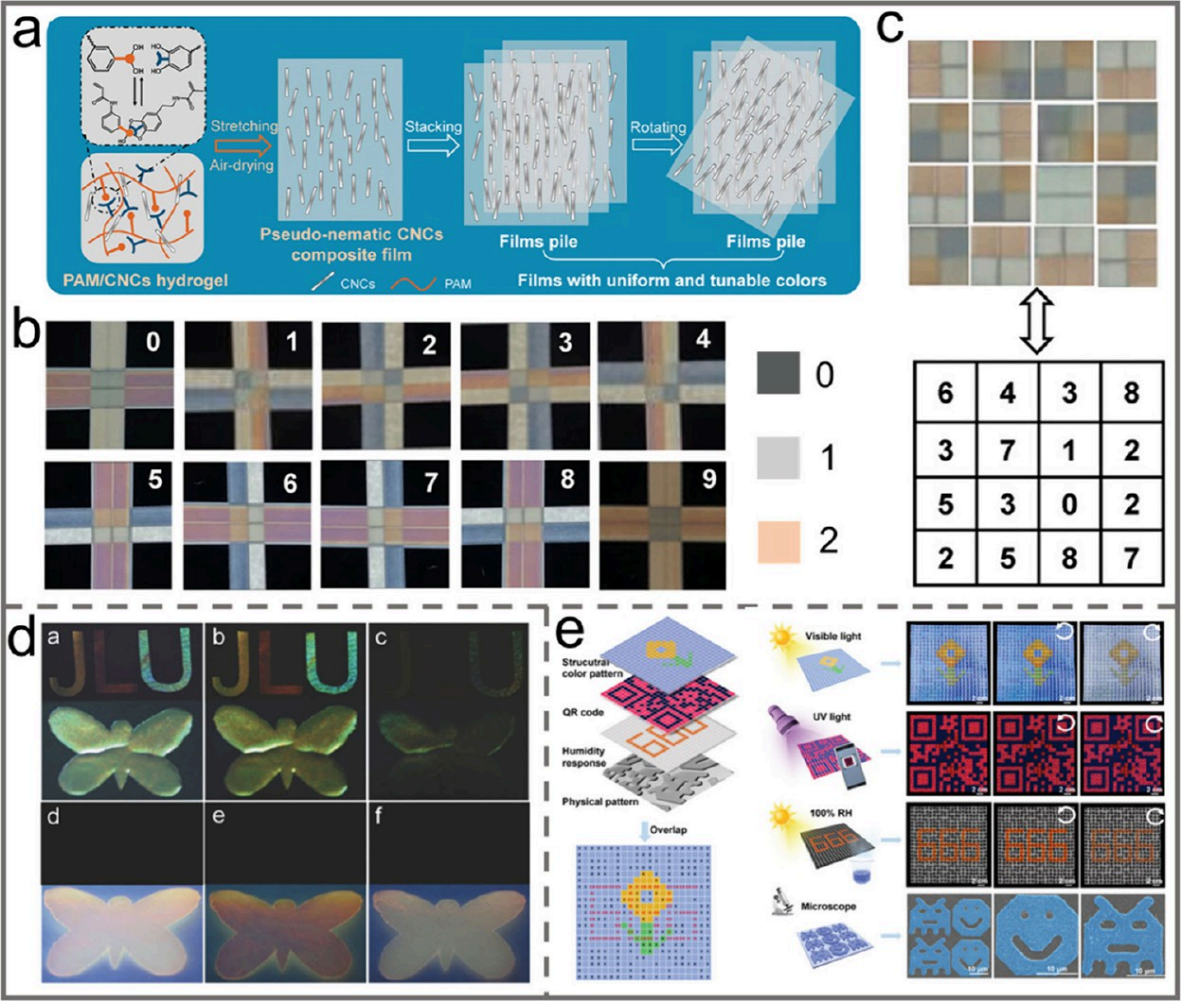
a) Schematic diagram showing the fabrication of CNC composite films with uniform and tunable colors. b) Ten fundamental elements, representing 0–9 decimal, were formed by stacking patterned composite films between the crossed polarizers. c) A 3D code for information storage and encryption through the combination of fundamental elements. Reprinted with permission from ref (8). Copyright 2020 Wiley-VCH. d) The potential of chiral photonic CNC films for polarization-based encryption. Reprinted with permission from ref (21). Copyright 2018 Wiley-VCH. e) Integrated five-channel encryption of CNC/bioAuNCs biolabels. Reprinted with permission from ref (9). Copyright 2023 Wiley-VCH.

Yang et al. developed an optical encryption system based on polyacrylamide (PAM)/CNC composite films.^8^ The composite films containing highly ordered CNCs were fabricated via mechanical stretching, followed by air drying of the dynamic-bond cross-linked PAM hydrogel (Figure 19a). Of note, CNCs within the hydrogel were prealigned via mechanical stretching. After that, the surface tension during the air-drying process further improved the orientation of the nanocrystals, resulting in the formation of the nematic CNC composite films. Owing to the light retardation resulting from the oriented CNCs, vivid interference colors presented by the composite films could be observed. The composite films were employed as modular components and piled together in certain thicknesses and rotation angles to realize the appearance of desirable interference colors. An optical ternary-coded decimal system was developed based on the as-prepared piled composite films. In this system, each decimal numeral of 0–9 was represented by the four colorful squares (Figure 19b). As shown in Figure 19c, 3D QR codes containing desirable digital information were successfully constructed with the CNC film piles. Xu et al. reported that chiral nematic CNC films fabricated by the EISA approach enabled left-handed and right-handed passive CPL (passive L-CPL and R-CPL), and right-handed CPL emission because of its left-handed helical structure and photonic bandgap (PBG).^21^ Moreover, the passive CPL was possible in the region ranging from near-UV to near-IR. By coassembling with achiral luminophores, the resulting photonic composite films with desirable R-CPL emission could be obtained. Notably, the R-CPL emission could be tuned by selecting the types of luminophores and tailoring the PBG. Given the intrinsic CPL ability of the photonic CNC-based films, they showcased the potential of neat CNC films and CNC/luminophores composite films in encryption. As shown in Figure 19d, the designed pattern consisted of JLU-shaped photonic neat CNC film and butterfly shaped CNC/luminophores composite film. Of note, the CNC composite film contained two types of organic luminophores, i.e., M1 emission at 512 nm and M3 emission at 585 nm, which were inside and outside the PBG, respectively. M1 contributed to the modulation of the R-CPL emission, while M3 was responsible for the tuning of the film color under irradiation. When observed under natural light against a black background, the iridescent color of the JLU-butterfly pattern was easily discernible to the naked eye. Moreover, the colors appeared more vibrant and subdued when viewed through left- and right-polarizing filters, respectively. Under 365 nm irradiation, “JLU” was nonemitting, while the combination of the right-circularly polarized (R-CPL) emission from M1 within the photonic bandgap (PBG) and the spontaneous emission from M3 resulted in the reddish beige color of the “butterfly”. Thus, the “JLU-butterfly” pattern was observed as “butterfly”. By observation of the “butterfly” pattern through left-polarizing and right-polarizing filters, distinct color variations were observed. When viewed through a left-polarizing filter, the “butterfly” took on an orange hue, predominantly due to the left-circularly polarized (L-CPL) emission from M3. Conversely, when observed through a right-polarizing filter, the “butterfly” exhibited a beige color, primarily attributed to the combined R-CPL emissions from both M1 and M3. Given that such a simple design utilizing neat photonic CNC film and CNC composite film can lead to diverse optical patterns, it is believed that a powerful system with multiple channels based on structural-color CNC or ChNC materials is possible for encryption/anticounterfeiting applications.

Chiroptical biolabels with multiple channels were constructed by the host–guest self-assembly of chiral CNCs and photoluminescent bovine serum albumin (BSA) stabilized-gold nanoclusters (AuNCs) (Figure 19e).^9^ The chiral nematic CNCs and the well-organized AuNCs immobilized on their surfaces were responsible for the structural color and photoluminescent color with strong circularly polarized luminescence (CPL) activity, respectively. The resulting CNC/AuNC composite films enabled anticounterfeiting and complex information encryption through multidimensional control of the polarization and lighting environment. Considering the fact that Cu^2+^ can competitively coordinate with BSA, while l-ascorbic acid (VC) exhibits stronger coordination and redox reaction with Cu^2+^ than BSA, the reversible PL was achieved by the addition of BSA and VC, which enabled encoding/erasing rewriting capability in the photoluminescent channel. Meanwhile, Ca^2+^-induced cross-linking of CNC templates allowed the humidity-responsive patterning in structural color channels. Moreover, customized micro/nano physical patterns that are only visible in microscopy were integrated onto the surface of the biolabels via self-assembling CNC/AuNC at the predesigned topographical patterned templates, which greatly extended information encryption capability. Although different types of CNC-based photonic encryption/anticounterfeiting systems have been developed, considerable efforts are still required to promote the practical application of such promising materials.

### 5.3 Templates for Chiral Mesoporous Materials

Functional materials that combine photonic structures and porosity have been actively pursued, owing to their significant potential in chiral sensing/recognition, chiral separation, and stereospecific catalysis. The chiral nematic self-assembling behaviors of CNCs and ChNCs, as well as their nanosized dimensions and high surface area, make such nanocrystals promising templates for constructing various types of chiral nematic mesoporous materials.

**Figure 20:**
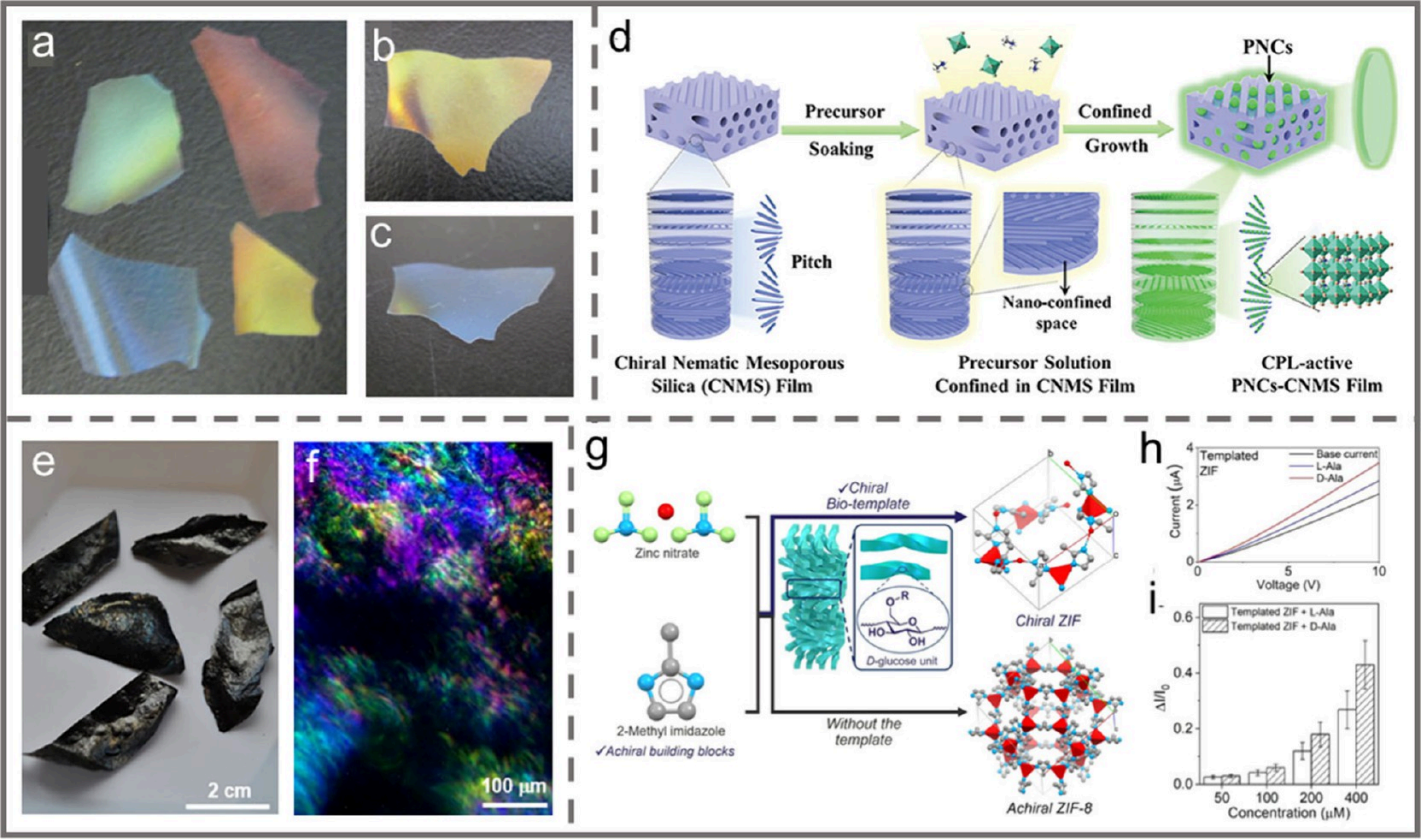
a) Photograph of mesoporous silica films with different colors. b–c) Photograph of a mesoporous silica film taken at different incidences. Reprinted with permission from ref (10). Copyright 2010 Springer Nature. d) Schematic diagram of in situ confined growth of PNCs within CNC film. Reprinted with permission from ref (176). Copyright 2024 Wiley-VCH. e) Photo of TiC film. f) PLM image of the TiC film. Reprinted with permission from ref (177). Copyright 2019 American Chemical Society. g) Schematic illustration of the production of templated ZIF. h) Typical I–V curve of templated ZIF to l-Ala and d-Ala at 400 μM. i) Current change (ΔI/I_0_) plot of templated ZIF at different l- and d-Ala concentrations from 50 to 400 μM. Reprinted with permission from ref (178). Copyright 2023 Wiley-VCH.

The idea of synthesizing mesoporous silica films by sol–gel mineralization using CNC as a template was reported in 2003. Dujardin et al. found that aqueous CNC suspensions could be mixed with prehydrolyzed tetramethoxysilane (TMOS) solutions. Birefringent silica replicas exhibiting patterned mesoporosity were obtained after the removal of CNC templates by calcining at 400 °C for 2 h.^179^ However, long-range chiral nematic structures were not found in this material. Until 2010, free-standing chiral nematic mesoporous silica films with long-range chiral nematic structures imparted by CNCs were fabricated by MacLachlan et al. (Figure 20a–c).^10^ The high surface area and chiral nematic structure of CNCs were precisely replicated in an inorganic solid. The reflected wavelengths of the films can be varied in the visible and near-infrared spectral ranges by changing the silica precursor-to-CNC ratio. Note that owing to their mesoporosity and chiral nematic structures, such chiral nematic mesoporous materials, inherited from self-assembled CNC, have emerged as promising chiral mesoporous hosts to combine achiral luminescent materials for the synthesis of circularly polarized luminescence (CPL)-active materials. For instance, chiral mesoporous silica was successfully utilized as a host to allow the in situ growth of various achiral halide perovskite nanocrystals (PNCs) (Figure 20d).^176^ The resulting composite displayed stable CPL emissions with full-color tunability and remarkable luminescent dissymmetric factors reaching up to −0.17.

More recently, besides chiral nematic mesoporous silica, considerable efforts have been devoted to synthesizing many other types of materials with chiral nematic mesoporous structures, including metal, metal oxides, and polymers, just to name a few, by taking advantage of the nanoscale and the chiral nematic order of the CNC templates. For instance, by using a similar sol–gel process, mesoporous TiO_2_ films containing replicas of the chiral nematic structures were synthesized and utilized as photocatalysts for phenol degradation and H_2_ production.^180^ Controlling the amount of d-glucose added into the CNC suspension resulted in the formation of chiral nematic structures with different helical pitches, thus finally leading to the photonic TiO_2_ films with the desired stopband. Compared to mesoporous TiO_2_ without chiral nematic structures, the obtained mesoporous TiO_2_ films containing replicas of the chiral nematic structures showed improved kinetics of H_2_ production and phenol photocatalytic degradation, as well as enhanced TiO_2_ photoconductivity. To further enhance the photoefficiency, various types of metal oxides including copper oxide, vanadium oxide, nickel oxide, and bismuth oxide, could be easily coupled the lamellar mesostructured TiO_2_ via a straightforward self-assembly method, resulting in higher H_2_ generation. Peroxotitanates (PTs), a kind of anionic, water-soluble complexes, were reported to coassemble with CNCs to fabricate peroxotitanate/CNC composites with tunable colors.^177^ After hydrothermal treatment followed by calcination in air, the obtained peroxotitanate/CNC composites could be transformed into robust, semitransparent TiO_2_ films with layered mesoporous structures. Additionally, freestanding mesoporous TiC films with chiral nematic order were successfully obtained by the magnesiothermic reduction of carbonized peroxotitanate/CNC assemblies (Figure 20e and f). The mesoporous TiC films showed excellent capacity retention performance, which may serve to develop freestanding anode materials for rechargeable lithium-ion batteries with long-term stability.

Chiral metal–organic frameworks (MOFs), as ordered porous materials with great potential for chiral catalysis, sensing, and enantiomers, have attracted considerable attention. The conventional approach to synthesizing chiral MOFs involves utilizing chiral organic molecules as primary linkers or auxiliary ligands. However, this method presents a significant limitation, as it often necessitates the use of costly and specialized chiral building blocks, which are available from a restricted selection of sources. Recently, Tsukruk et al. reported a scalable and effective method for achieving chiral zeolitic imidazolate framework (ZIF) MOF, unc-[Zn(2-MeIm)2,2-MeIm = 2-methylimidazole], from achiral precursors by utilizing chiral nematic organization of CNCs as hierarchical biotemplates (Figure 20g).^178^ CNCs were selected as biotemplates due to their three levels of chirality: (i) asymmetric carbon atoms in d-glucose units at the molecular level; (ii) spindle-shaped nanocrystals at the nanoscale; and (iii) left-handed chiral nematic liquid crystal organization at the macroscale. The templated chiral ZIF demonstrates chiral sensing and enantioselective recognition abilities with a low detection limit of 39 μM and a chiral detection limit of 300 μM for the chiral amino acids d- and l-alanine (Figure 20h and i). The chiral nematic CNC suspension, beyond serving as templates, can be transformed into free-standing cellulosic aerogels through supercritical CO_2_ drying.^181^ Subsequent carbonization under an Ar atmosphere resulted in the formation of free-standing activated carbon aerogels with a hierarchical order derived from the chiral nematic organization of CNCs. These activated carbon aerogels exhibited interconnected meso/micropores and boasted specific surface areas of up to 820 m^2^ g^–1^. Notably, they also displayed superparamagnetic behavior with a maximum magnetization value of 17.8 ± 0.1 emu g^–1^. Depending on the composition, such aerogels possessed compressive modulus values ranging from 179 to 400 kPa. When utilized as symmetric supercapacitor electrodes, the activated carbon chiral nematic aerogels showcased an impressive specific capacitance of 294 F g^–1^, maintaining 92.8% capacitance retention after 2500 cycles at a scan rate of 50 mV s^–1^.

**Figure 21:**
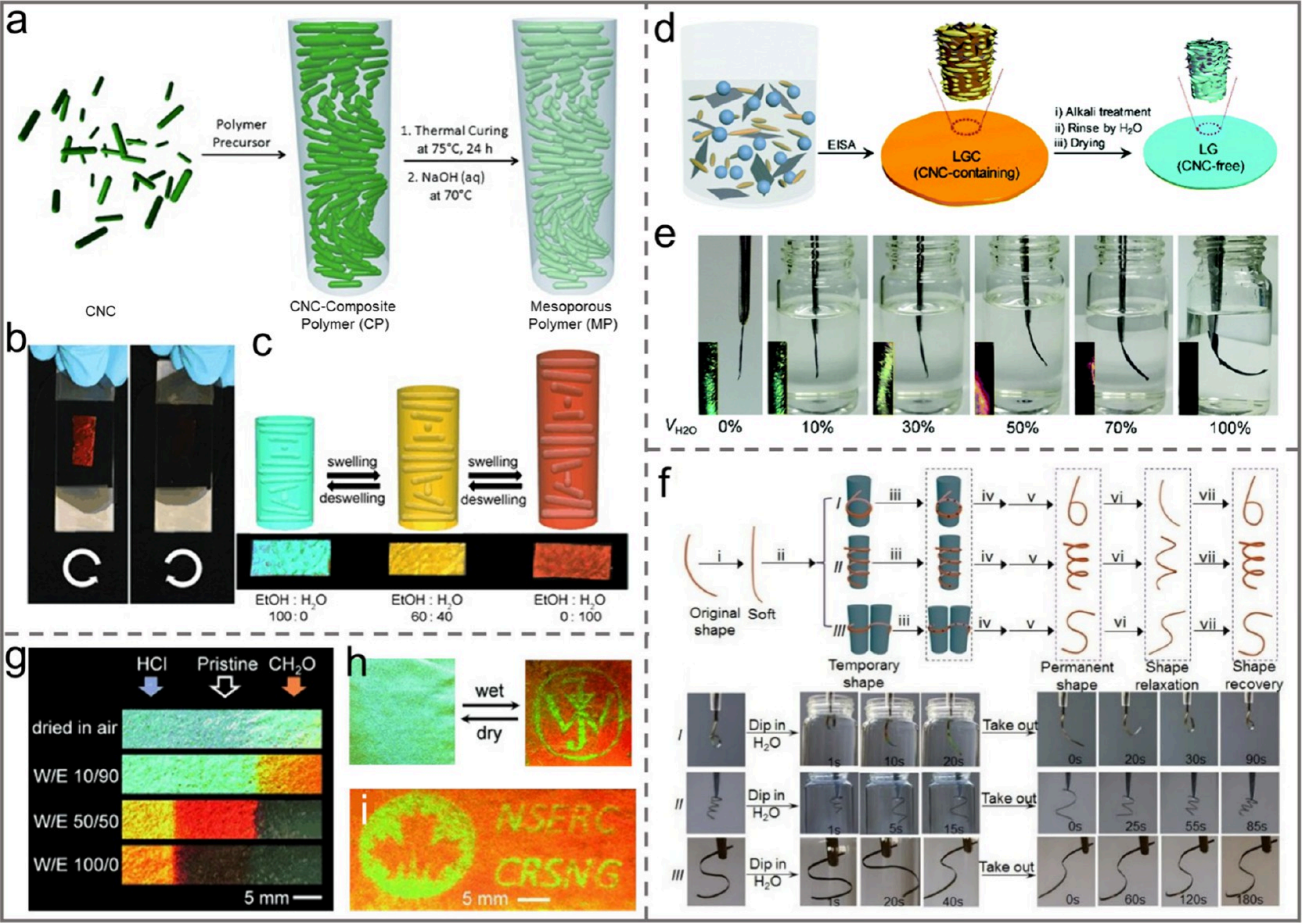
a) Schematic diagram showing the synthesis of mesoporous chiral nematic phenol-formaldehyde resins. b) Photographs of a sample under left-handed circular polarizer (left) and right-handed polarizer (right). c) Schematic illustration (top) and photographs (bottom) of the samples in water and ethanol mixtures. Reprinted with permission from ref (182). Copyright 2013, Wiley-VCH. d) Schematic illustration of the fabrication of latex-GO composite film. e) Photographs of the film strips immersed in the mixtures of H_2_O/*n*-PrOH with different VH_2_O. Reprinted with permission from ref (183). Copyright 2019 Royal Society of Chemistry. f) Illustration and photographs of the film strips with predetermined shapes treated by FoA and their responses to the wetting-drying circle. Reprinted with permission from ref (184). Copyright 2019 Royal Society of Chemistry. g) Photographs of mesoporous polymer resin films in a mixture of water and ethanol with different proportions. h) Complicated image patterned on a mesoporous film, and the pattern can be revealed by swelling in water. i) Complicated image patterned on a mesoporous film, and the pattern can be revealed by swelling in 20/80 (v/v) water/ethanol mixture. Reprinted with permission from ref (185). Copyright 2015, Wiley-VCH.

The CNC templating strategy was additionally employed to create chiral nematic mesoporous polymers. It is important to note that, compared to extensive research on inorganic mesoporous materials synthesized through chiral nematic CNC templates, there has been significantly less investigation into the fabrication and application of organic mesoporous materials using chiral nematic CNC organizations. Khan et al. reported a chiral mesoporous phenol-formaldehyde (PF) resins by using CNCs as templates (Figure 21a).^182^ The color of the composite films can be tuned by varying the ratio of the PF precursor to CNC or the ionic strength of the mixture. Flexible mesoporous polymer films with photonic properties were obtained after the CNC template was removed by NaOH treatment. These films appeared bright red under a left-handed circular polarizer while turning to dull brown under a right-handed circular polarizer (Figure 21b). Moreover, the films showed only a slight color change after immersing in anhydrous ethanol. In contrast, polymer films immersed in ethanol/water mixtures and pure water displayed obvious, tunable color changes. Furthermore, the systematic changes in color can be easily observed by the naked eye when swelling in ethanol/water mixtures with different proportions (Figure 21c). These mesoporous films have potential applications in sensing and optics due to their flexibility, photonic properties, and responsive swelling behavior. Then, the same group reported a bilayer chiral nematic resin film with tunable optical properties and rapid mechanical response.^186^ The bilayer film was fabricated by using two mesoporous phenol formaldehyde (PF) resin layers containing CNCs-based chiral nematic structures with different helical pitches. Such a mesoporous PF resin bilayer film with chiral nematic structures can selectively reflect light of two different wavelengths. Moreover, the differential swelling behaviors of the two layers caused the thin films to curl after exposure to different solvents. The stimulation of reactive actuation combined with the changes in photonic properties makes these materials attractive in the field of optics and soft-body robotics.

Actuators with a bilayer structure often suffer from weak interlayer connections, leading to detachment after repeated use. Yuan et al. recently reported the fabrication of flexible mesoporous latex/GO composite films with a single-layer structure, templated by CNCs through EISA of an aqueous dispersion containing latex, GO, and CNCs (Figure 21d).^183^ Driven by gravitational differences and entropy increases, GO distributed unevenly, predominantly gathering at the top of the film. This asymmetrical organization imparts different wettabilities to the top and bottom surfaces of the film. Furthermore, the chiral nematic order was retained within the film after the removal of CNC via NaOH treatment. As a result, the film exhibited synchronous color and actuation responses (Figure 21e).

The aforementioned mesoporous films generally exhibit simple bending and curling movements due to their undesigned shapes. To achieve more sophisticated motions, mesoporous PF resin/GO-based actuators with predesigned shapes were fabricated via the standard EISA approach.^184^ Similar to latex/GO composite films, the obtained mesoporous resin film exhibited a GO-depleted top surface and a GO-rich bottom surface and displayed structural colors derived from the internal chiral nematic architecture. By selectively treating the films with aldehydes followed by thermopolymerization, objects formed from fully wetted film bars can achieve various permanent shapes and exhibit robust shape recovery behavior over multiple wetting-drying cycles (Figure 21f).

Dynamic photonic patterns were fabricated with mesoporous phenol-formaldehyde resins.^185^ After swelling in polar solvent, the mesoporous PF resins showed red-shift which can be tuned by postsynthetic treatment with acid or formaldehyde (Figure 21g). With acidification, the cross-linking of the films increased, leading to a decrease in the concentration of methylol groups on the surface of the films and a decrease in hydrophilicity. Conversely, formaldehyde treatment increased the density of methylol groups, thereby increasing the hydrophilicity. After treatment, the resin exhibited different swelling behaviors, leading to changes in chiral nematic pitch and reflected color. Thus, acid and formaldehyde could be used as inks to write on the films, generating latent images that appear only when the film swells (Figure 21h and i).

Inorganic optical mesoporous materials have also been successfully prepared by ChNC-induced ordering self-assembly of inorganic particles. Kato and co-workers fabricated anisotropic ChNC-templated calcium carbonate (CaCO_3_) composite materials.^187^ First, free-standing ChNC gel films were obtained by soaking the concentrated liquid crystalline solution (15 wt %) in methanol. Then, the gels were manually stretched to further align the nanocrystals. The obtained ChNC films were used as templates for the growth of CaCO_3_. It was found that rod-shaped CaCO_3_ crystals grew along the long axis of the ChNC backbone within the stretched films. However, no rod-shaped crystals were obtained when unstretched ChNCs films were used as the matrix. ChNCs/CaCO_3_ composite films with helical structures were prepared by a biomineralization-inspired crystallization process.^188^ Helical chitin/PAA templates were synthesized from a chiral nematic liquid crystalline ChNC suspension, which were then immersed in the colloidal CaCO_3_ suspension at room temperature. Transparent films were obtained after complete drying, in which the weight fraction of CaCO_3_ increased from 12 wt % after 1 day of deposition time to 23 wt % after 7 d.

Mesoporous silica materials were obtained after calcination of nanocomposites, and the porosity was strongly correlated to the initial concentration of ChNC. When using ChNCs as templates to prepare mesoporous silica materials, the porosity and chiral nematic structure of films were determined by the initial concentration of ChNC.^189^ Though layered nematic structures in the films were confirmed by TEM, no chiral nematic structure was found. Nguyen et al. reported the preparation of mesoporous silica and organosilica films with layered structures and high surface areas using ChNCs as templates.^190^ ChNC templates were removed by calcination or sulfuric-acid-catalyzed hydrolysis to yield large, crack-free mesoporous silica and ethylene-bridged organosilica films. Mesoporous nitrogen-doped carbon materials with layered structures were fabricated using ChNC as a soft template by a three-step method.^191^ ChNCs were first encapsulated within sol–gel silica; then, the resulting silica/ChNC composite was carbonized, and the silica was etched. The resultant mesoporous nitrogen-doped carbon films can be used as electrode materials for supercapacitors.

### 5.4 Self-Assembly in Confined Geometry

Recently, the self-assembly of CNCs and ChNCs in confined spaces such as tubular and spherical geometries has established itself as an efficient way to construct chiral nematic materials that possess properties distinct from those fabricated in planar unconfined geometry. Of note, in comparison to self-assembly in unconfined space that has been widely explored, the self-assembly of CNCs/ChNCs under confinement is still in its infancy.

**Figure 22:**
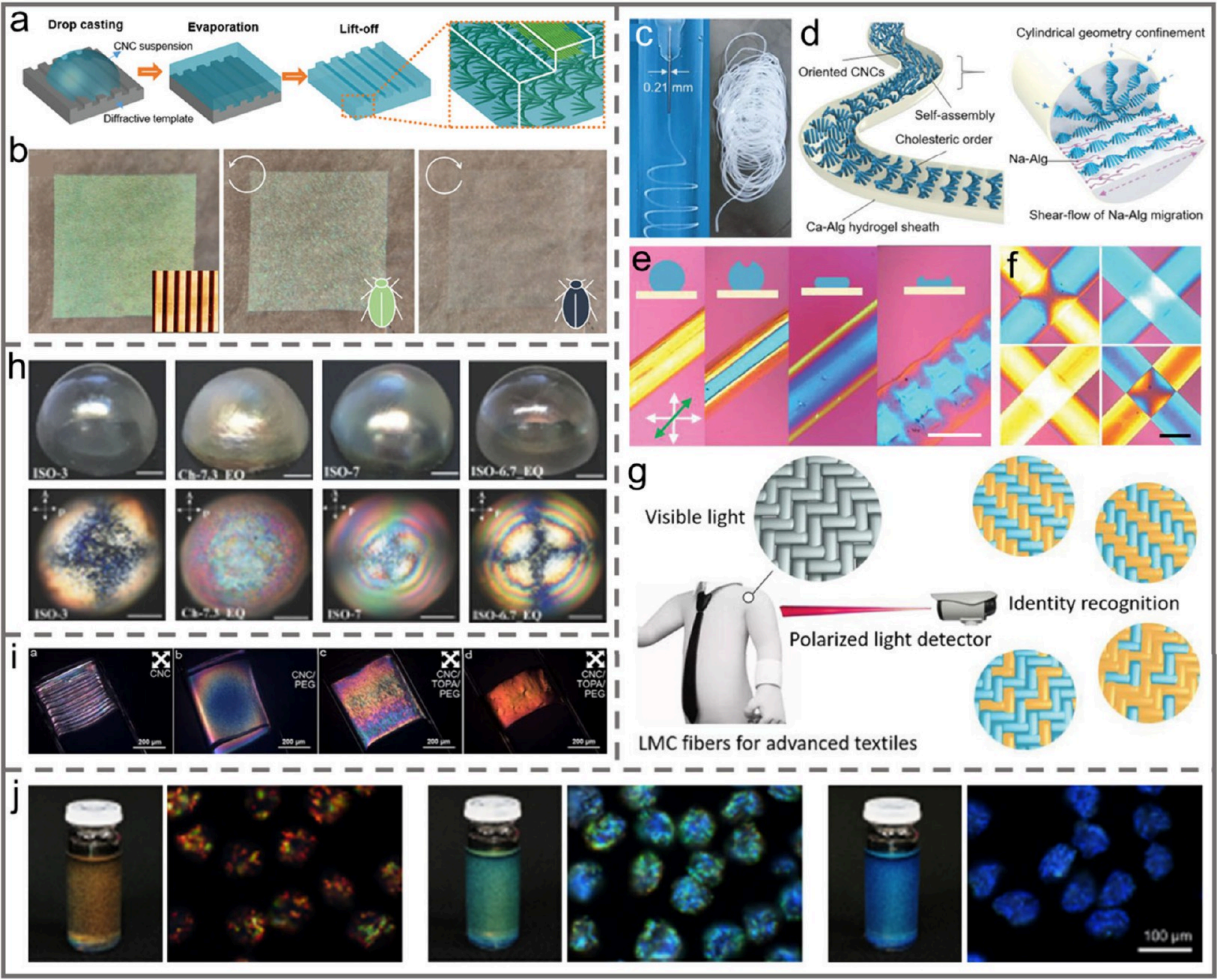
a) Schematic illustration of the self-assembly of chiral CNC photonic structures with surface gratings. b) Photographs of CNC film patterned with the grating periodicity of 1.6 μm under no polarizers (left), left-hand circular polarizer (middle), and right-hand circular polarizer (right). Reprinted with permission from ref (192). Copyright 2019 Wiley-VCH. c) Fabrication of scalable LMC fibers. d) Schematic illustration showing the formation of the chiral nematic liquid crystalline phase within the LMC fibers and the mechanism of the self-organization of LMCs in a dynamically confined, cylindrical geometry. e) PLM-λ images of the LMC fibers with different optical appearances by applying external stress. f) PLM-λ images of two crossed LMC fibers showing different combinations of optical appearances at the position of right-rotated 45°. g) Schematic illustration of the advanced fabrics showing different colors for identification. Reprinted with permission from ref (12). Copyright 2020 Wiley-VCH. h) Photographs of four curved films with a radius R = 2 mm (top) and the corresponding polarized optical microscopy images of these films (down). Reprinted with permission from ref (193). Copyright 2018 Wiley-VCH. i) PLM images of pure and composite CNC films drying in the rectangular capillaries. Reprinted with permission from ref (194). Copyright 2021 American Chemical Society. j) Photographs of cellulosic photonic pigments. Reprinted with permission under a Creative Commons Attribution (CC BY) License from ref (13). Copyright 2022 Springer Nature.

Xiong et al. explored the combination of optical structures and 3D chiral arrangement in CNC films by patterning the surface of CNC films with different templates.^192^ The surface-patterned CNC films displayed ordered chiral nematic structures inside the film and highly oriented CNC gratings on the topmost embossed grooves (Figure 22a). Compared with traditional uniform CNC films, the patterned films exhibited narrower selective reflection width, high CPL, and a view-angle-dependent color appearance (Figure 22b). Utilizing the same method, Chu et al. developed a hierarchically structured paper with dual photonic structures, including the interior chiral nematic organizations and the periodic grating lines on its surface.^195^ The patterned photonic films were fabricated by confining CNC suspensions in highly aligned microgrooves via soft nanoimprinting lithography and EISA processes. After drying, the films with highly ordered grating lines on their underside were peeled off of the template. Liu et al. developed a simple microfluidic strategy to produce hierarchical liquid metacrystal (LMC) fibers with core–sheath structures through the confined self-assembly of CNC within a hydrogel sheath formed by the fast gelation of sodium alginate (Na-Alg) and Ca^2+^ ions (Figure 22c).^12^ 3D topological architectures consisting of long-range chiral nematic liquid crystalline configurations were formed within the LCM fibers, including radial topological structures and axial awl-shaped topological structures. The sheath layer played an important role in preventing shape deformation and providing a continuous and stable self-assembly environment (Figure 22d). During the drying process, the shape of LMC fibers could be reconfigured by applying external stress, resulting in a highly controlled color distribution (Figure 22e). The obtained LMC fibers not only displayed vivid, tunable interference colors and inverse optical activity but also had the ability to precisely tune linearly and CPL in a half-sync/half a sync form (Figure 22f). The obtained fibers showed potential applications in the field of advanced textiles with different colors for identification (Figure 22g).

Rofouie et al. reported the production of semispherical photonic chiral nematic films through the confined self-assembly of CNC in semispherical cavities (Figure 22h).^193^ The optical properties and structure of the resulting films can be tuned by changing the cavity curvature, the composition of the precursor CNC suspension, and its liquid crystalline or isotropic state. The spherical shape of the films facilitated polydomain structure formation and helix axis inclination, thereby producing a broadband reflection spectrum. Crack-free, uniform, and monolithic thin photonic films were produced through confined self-assembly of CNC in rectangular capillaries (Figure 22i).^194^ CNC, PEG, and 3,4,5-trihydroxyphenethylamine hydrochloride (TOPA) were used as the liquid crystalline phase material, polymer plasticizer, and adhesion promoter, respectively. It was found that no cracks formed for CNC/TOPA/PEG composite film, while numerous cracks formed for pure CNC film and few cracks formed for CNC/PEG composite film. The improved hydrogen bonding achieved by the addition of TOPA and the relaxation of the internal stress with the assistance of PEG jointly suppressed the formation of cracks for the CNC/TOPA/PEG composite film. Moreover, the chiral nematic structure propagated through the entire thickness and across centimeter-sized surface areas, enabling the obtained uniform, crack-free films with vivid interference colors and improved toughness as well as the ultimate tensile strength. Photonic pigments that reflect colors across the entire visible spectrum were produced by confined self-assembly of chiral nematic CNC suspension within emulsified microdroplets (Figure 22j).^13^ During the drying process, the droplets underwent numerous buckling events, which made the shrinkage of the nanostructure greater than that predicted for spherical geometries. The interfacial buckling distorted the chiral nematic structure of CNCs, resulting in both LCP and RCP light reflections. Furthermore, the cellulose photonic pigments integrated into a matrix displayed an angle-independent structural color. Liu et al. reported the confined self-assembly of ChNCs in the capillaries.^28^ In the confined environment, the air–liquid interface at the end of the capillary served as both the evaporation interface and the initial deposition site of ChNCs, which was different from the moving evaporation interface in the previous EISA modalities. The formation of tactoids was suppressed during the whole self-assembly process, while birefringent ChNC multilayers with nested multiple paraboloid structures and a density gradient were gradually generated, finally resulting in the formation of cylindrical ChNCs-based photonics.

### 5.5 Potential Strategies for Large-Scale Production of PNs-Based Chiral Nematic Materials

**Figure 23:**
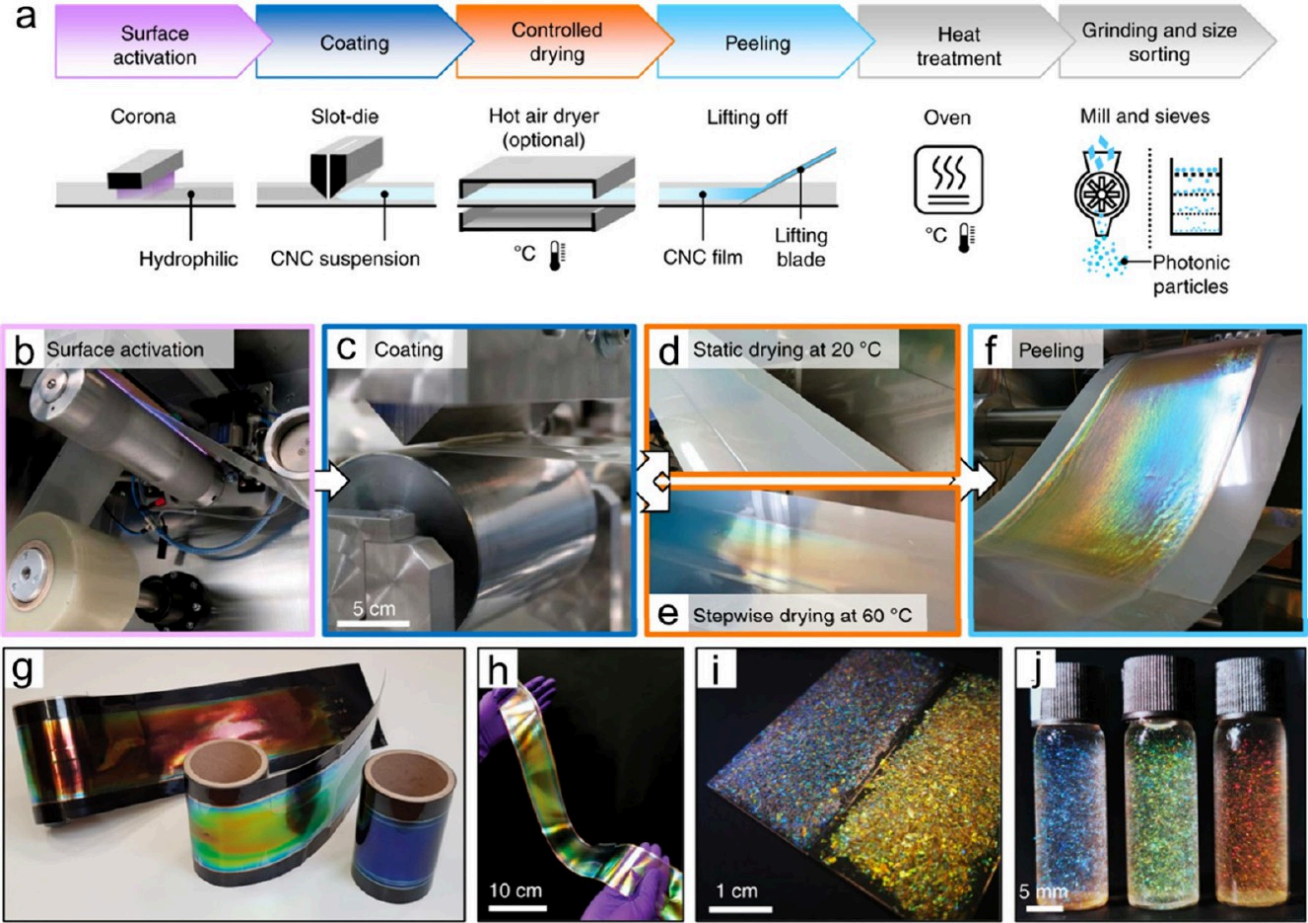
a) Flowchart describing the key steps in the fabrication of photonic CNC particles. b) Photograph of the corona etching step. c) Photograph of the slot-die coating of CNC suspension onto the web. d) Photograph of static drying at 20 °C. e) Photograph of continuous progressive drying at 60 °C. f) In-line peeling of a CNC film from the web. g) CNC films deposited onto a black PET web. h) Photograph of a free-standing R2R-cast CNC film. i) Untreated (left) and heat-treated (right) photonic CNC particles embedded in transparent varnish before size sorting. j) Heat-treated photonic CNC particles (pigments) immersed in ethanol and 50% aqueous ethanol (from left to right). Reprinted with permission from ref (196). Copyright 2022 Springer Nature.

The traditional methods for fabricating chiral nematic CNC films, such as EISA, often require several hours or even days and are limited to small-scale production, hindering their industrial application. Thus, there is a significant demand for simple and efficient methods to enable the continuous production of large-scale chiral nematic films. As introduced above, Atifi and colleagues reported a rapid and scalable technique using electrodeposition to create chiral nematic CNC films with long-range order in just a few minutes.^138^ Although this approach demonstrated the ability to prepare chiral nematic CNC films within a short time, the possibility of fabricating large-scale photonic films via this method was not verified. Vignolini et al. presented an industrially relevant method to scale up the production of structurally colored films by casting a commercially available CNC suspension on a commercial roll-to-roll (R2R) coating unit (Figure 23).^196^ The formulation of the CNC suspension and the deposition and drying conditions were extensively studied to enable the production of flexible structural CNC films. Notably, to demonstrate the continuous production of photonic CNC films, the R2R process was modified to include an in-line hot-air dryer to accelerate evaporation and stepwise translation to reduce the effective deposition speed of the web. After heating at 180 °C for 30 min, the prepared films could be ground into particles suitable for use as effect glitter and pigments, providing an eco-friendly alternative to nonbiodegradable microplastics and unsustainable inorganic pigments. Not only that, despite the growing demand for chiral nematic films derived from PNs, progress in developing suitable approaches for their large-scale production has been limited. Concerted efforts should be devoted to advancing technologies for the large-scale production of chiral nematic films in the near future.

## 6 CONCLUSIONS AND OUTLOOK

Chiral nematic architectures featuring special functionalities have been found in various living systems on different scales. Replicating such structures and exploring their fascinating properties and broad applications have been and continue to be highly pursued. With merits of abundance, intrinsic high biocompatibility and biodegradability, and ease of modification, PNs, in particular, CNCs and ChNCs, are capable of self-organizing into chiral nematic structures, making them ideal nanobuilding blocks for constructing various functional biomimetic chiral nematic materials.

This review intends to summarize the latest advances and current knowledge regarding the self-assembly of CNCs/ChNCs into chiral nematic structures, highlighting the introduction of cellulose- and chitin-based chiral nematic structures in living organisms, various crucial factors affecting the chiral nematic structures formed via self-assembly of CNCs and ChNCs as well as the optical properties of the self-assembled materials. The application of the CNCs- and ChNCs-based materials containing chiral nematic structures in stimuli-responsive photonic sensors, photonic encryption, and templating for chiral mesoporous materials have also been described. Moreover, a largely underexplored area, that is, self-assembly of these nanocrystals under confinement, has also been summarized.

While significant progress has been achieved during the past decades in the aspects of unveiling self-assembly mechanisms, easing fabrication procedures, and broadening applications, there are still some grand challenges for large-scale construction and application of CNC- and ChNC-based chiral nematic materials. First, the realization of uniform structural colors within large-scale CNC- and/or ChNC-based functional materials is of paramount significance but extremely challenging owing to the difficulty of precisely controlling the alignment of chiral nematic domains and the helical pitch during the self-assembly process. Some recent research findings, for instance, the visualization of the structural evolution of CNC tactoids to chiral nematic films, may contribute to the preparation of high-quality photonic materials with uniform structural color. Second, time-saving, effective, and feasible approaches that can accommodate large-scale and continuous production of chiral nematic materials are highly required. For most self-assembly approaches, for instance, EISA, the long time required for film formation is a major hurdle to the large-scale production of CNC/ChNCs-based chiral nematic materials. Notably, the self-assembled materials prepared by EISA showed poor repeatability. Third, the precise mechanism lying behind the self-assembly process or, in other words, the driving force in the self-organization of CNCs/ChNCs into chiral nematic structures is not yet clear. Multiple advanced characterization techniques, e.g., in situ synchrotron X-ray scattering and cryogenic electron microscopy, combined with theoretical and rational modeling, could offer rich possibilities for a deeper understanding of the self-assembly mechanism.

Overall, despite the great strides that have been fulfilled in the area of self-assembly of CNCs and ChNCs into chiral nematic architectures, this research realm is still in its infancy. We believe that interdisciplinary communication and collaboration are key to future research and the acceleration of the development of chiral nematic PNs-based products and technologies.

## VOCABULARY

Polysaccharide nanocrystals: crystalline regions derived from polysaccharides such as cellulose nanocrystals and chitin nanocrystals, which are large carbohydrate molecules composed of repeating sugar units.
Cellulose nanocrystals: the crystalline components isolated from cellulose at the nanoscale.
Chitin nanocrystals: the crystalline components isolated from chitin sources at the nanoscale.
Self-assembly: a process in which molecules or nanoscale building blocks spontaneously organize into well-defined, stable structures without human intervention or guidance. This process is driven by various noncovalent interactions such as hydrogen bonding, van der Waals forces, electrostatic interactions, and hydrophobic effects.
Chiral nematic structure: a type of liquid crystal phase that exhibits a helical arrangement of molecules. This structure combines the properties of nematic liquid crystals, where molecules are aligned parallel to each other, with a chiral order, resulting in a helical pattern.
